# Molecular Mechanisms in Perirhinal Cortex Selectively Necessary for Discrimination of Overlapping Memories, but Independent of Memory Persistence

**DOI:** 10.1523/ENEURO.0293-17.2017

**Published:** 2017-10-18

**Authors:** Magdalena Miranda, Brianne A. Kent, Juan Facundo Morici, Francisco Gallo, Noelia V. Weisstaub, Lisa M. Saksida, Timothy J. Bussey, Pedro Bekinschtein

**Affiliations:** 1 Laboratory of Memory Research and Molecular Cognition, Institute for Cell Biology and Neuroscience CONICET and University of Buenos Aires Medical School, Buenos Aires, Argentina; 2 Systems Neuroscience Group, Laboratory of Experimental Cognition and Behavior, Institute of Physiology and Biophysics, IFIBIO “Houssay,” CONICET and University of Buenos Aires Medical School, Buenos Aires, Argentina; 3 Department of Medicine, University of British Columbia, Vancouver, BC, Canada; 4 Department of Psychology and MRC/Wellcome Trust Behavioural and Clinical Neuroscience Institute, University of Cambridge, Cambridge, UK; 5 Molecular Medicine Research Group, Robarts Research Institute, and Department of Physiology and Pharmacology, Schulich School of Medicine and Dentistry, Western University, London, ON, Canada; 6 The Brain and Mind Institute, Western University, London, ON, Canada

**Keywords:** Arc, BDNF, object recognition, pattern separation, perirhinal cortex

## Abstract

Successful memory involves not only remembering over time but also keeping memories distinct. The ability to separate similar experiences into distinct memories is a main feature of episodic memory. Discrimination of overlapping representations has been investigated in the dentate gyrus of the hippocampus (DG), but little is known about this process in other regions such as the perirhinal cortex (Prh). We found in male rats that perirhinal brain-derived neurotrophic factor (BDNF) is required for separable storage of overlapping, but not distinct, object representations, which is identical to its role in the DG for spatial representations. Also, activity-regulated cytoskeletal-associated protein (Arc) is required for disambiguation of object memories, as measured by infusion of antisense oligonucleotides. This is the first time Arc has been implicated in the discrimination of objects with overlapping features. Although molecular mechanisms for object memory have been shown previously in Prh, these have been dependent on delay, suggesting a role specifically in memory duration. BDNF and Arc involvement were independent of delay—the same demand for memory persistence was present in all conditions—but only when discrimination of similar objects was required were these mechanisms recruited and necessary. Finally, we show that BDNF and Arc participate in the same pathway during consolidation of overlapping object memories. We provide novel evidence regarding the proteins involved in disambiguation of object memories outside the DG and suggest that, despite the anatomical differences, similar mechanisms underlie this process in the DG and Prh that are engaged depending on the similarity of the stimuli.

## Significance Statement

In this article, we show, outside of the hippocampus, the molecular mechanisms underlying the ability to separate similar experiences into distinct memory representations (thought to result from the computational mechanism of pattern separation). The dentate gyrus (DG) is thought to disambiguate representations belonging to any domain, but other regions could also perform this operation. Although molecular mechanisms have been shown previously in the perirhinal cortex (Prh), these have always been dependent on delay, suggesting a role specifically in memory persistence. We report that, despite the profound anatomic differences between the perirhinal cortex (Prh) and the DG, the discrimination of overlapping memories in these regions relies on the same molecular mechanisms.

## Introduction

Two similar stimuli could be associated with two very different experiences: a cat inside your house may be friendly, whereas a puma could be threatening to your life. It is thought that the brain creates unique representations of similar events, which are less confusable and can be associated with different outcomes, through a process called pattern separation (Treves and Rolls, 1994; Gilbert et al. 1998; Leutgeb et al. 2007). The original computational models define the process in terms of a transformation of input representations into output representations that are less correlated with each other (Marr, 1971; Treves and Rolls, 1994; McClelland et al. 1995). Thus, pattern separation increases the likelihood of accurate encoding and subsequent retrieval. It has been studied effectively using electrophysiology (Leutgeb et al. 2007; Neunuebel and Knierim, 2014), and we and others have developed tasks to demonstrate the relevance of pattern separation processes to cognition (Gilbert et al. 1998; Kirwan and Stark, 2007; Clelland et al. 2009; Toner et al. 2009; Creer et al. 2010; Bekinschtein et al. 2013).

Because episodic memory involves the recollection of unique events, separation of similar experiences is proposed to be an essential component for the storage of nonconfusable representations of these episodes and has been studied mainly in the hippocampus (Ranganath, 2010). Indeed, the computational models focus specifically on DG granule cells, which are thought to be a domain-general pattern separator (Yassa and Stark, 2011), well suited for performing pattern separation on overlapping inputs from the entorhinal cortex. Adult neurogenesis in the DG has been shown to be required for discrimination of overlapping representations in the spatial domain (Gilbert et al. 1998; Clelland et al. 2009; Bekinschtein et al. 2014a), and some studies have begun to elucidate the molecular basis involved in this process (Bekinschtein et al. 2013, 2014b).

Because the hippocampus is known to mediate spatial memory in rodents, with the exception of a few studies (e.g., Johnson et al. 2017), most tasks used to evaluate the behavioral outputs thought to result from discrimination of overlapping representations in rodents have involved some kind of contextual or spatial manipulation (Gilbert et al. 1998; Clelland et al. 2009; Kheirbek et al. 2012; Nakashiba et al. 2012; Bekinschtein et al. 2013). However, this type of disambiguation could, in principle, occur during encoding of representations other than spatial, for example for objects in Prh (Kent et al. 2016). Indeed, disambiguation of object representations has been shown to require Prh (Bussey et al. 2002; Bartko et al. 2007), and it has been proposed that Prh discriminates similar objects by storing unique conjunctive representations of these items (Bussey et al. 2002; Bartko et al. 2007). However, it has been suggested that the DG is a domain-general discriminator of both spatial and object representations, among other types. Although molecular mechanisms have been shown previously in Prh, these have always been dependent on delay, suggesting a role specifically in persistence (Winters and Bussey, 2005b; Seoane et al. 2012). Manipulation of the Prh during acquisition or after learning produced delay-dependent effects on memory, but this does not indicate a specific effect on the ability to disambiguate similar input stimuli. It is not known whether a putative function of Prh in object disambiguation operates via the same molecular mechanisms as those shown within the DG (Bekinschtein et al. 2013).

In this work, we tested whether Prh is involved in the consolidation of overlapping object memories through plasticity-related mechanisms such as brain-derived neurotrophic factor (BDNF) that have been implicated during discrimination of overlapping spatial memories. We found that BDNF, a protein essential for memory storage (Bekinschtein et al. 2014a), is required for disambiguation of memories for similar objects in Prh, just as it is for spatial memories in the hippocampus. In addition, we found that activity-regulated cytoskeletal-associated protein (Arc), a molecule important for plasticity and memory (Bramham et al. 2010), is also required. This immediate early gene product has emerged as a key protein in memory formation and different types of synaptic plasticity, including long-term potentiation (LTP), long-term depression (LTD), and homoeostatic synaptic scaling (Bramham et al. 2010). Arc is strongly associated with neuronal activity related to behaviorally relevant experiences (Guzowski et al. 2005). In addition, this molecule has been shown to be required in various structures for different types of learning such as fear conditioning (Ploski et al. 2008) and inhibitory avoidance (Martínez et al. 2012). Arc-deficient mice present deficits in several learning tasks such as water-maze fear conditioning, conditioned taste aversion, and novel object recognition (Plath et al. 2006). This evidence points at Arc as a possible target of BDNF action. Finally we demonstrated that BDNF is likely to act upstream of Arc during the consolidation of “pattern-separated” object memories. We suggest that discrimination of similar, but not distinct, stimuli in the medial temporal lobe occurs not only in the DG, but also in the Prh, depending on the nature of the representations. Importantly, similar mechanisms underlie the discrimination of overlapping memories wherever it occurs, and these mechanisms are different from those that vary with demand on memory persistence.

## Materials and Methods

### Subjects

The subjects were 201 male Long-Evans rats from our breeding colony, weighing ∼250–300 g at the start o*f* testing. The rats were housed on a reversed 12-h light/12-h dark cycle (lights on 1900-0700), in groups of two or four. All behavioral testing was conducted during the dark phase of the cycle. Rats were food deprived to 85%–90% of their free feeding weight to increase spontaneous exploration, except during recovery from surgery, where food was available *ad libitum*. Water remained available *ad libitum* throughout the study. All experimentation was conducted in accordance with the National Animal Care and Use Committee of the University of Buenos Aires (CICUAL) and strict compliance with the guidelines of the University of Cambridge and United Kingdom Animals (Scientific Procedures) Act 1986 and the Amendment Regulations 2012.

### Surgery and cannulation

All rats were implanted bilaterally in Prh with 22-gauge indwelling guide cannulas. Subjects were anaesthetized with ketamine (Holliday; 74 mg/kg, i.p.) and xylazine (Konig; 7.4 mg/kg, i.p.) and placed in a stereotaxic frame (David Kopf Instruments) with the incisor bar set at −3.2 mm. Guide cannulas were implanted according to the following coordinates, measured relative to the skull at bregma (Paxinos and Watson, 1998): anteroposterior −5.5 mm, lateral ± 6.6 mm, dorsoventral −7.1 mm. The cannulas were secured to the skull using dental acrylic and three jeweler screws. Obturators, cut to sit flush with the tip of the guide cannulas and with an outer diameter of 0.36 mm, were inserted into the guides and remained there except during infusions. At the completion of each surgery, an antibiotic was applied for 3 d (enrofloxacin; 0.27 mg/kg, Vetanco). Animals were given at least 7 days to recover before drug infusions and behavioral testing.

### Infusion procedure

Depending on the experiment, rats received bilateral infusions of oligonucleotides (ODNs, 4 nmol/μl/0.5 μl side; Genbiotech), human recombinant BDNF (0.5 μg/μl/0.5 μl side; Byoscience), emetine (50 μg/μl/0.5 μl side; Sigma-Aldrich), or saline at different times during the behavioral task. The injection volume was always 0.5 μl/side. ODNs were HPLC-purified phosphorothioate end-capped 18-mer sequences, dissolved in sterile saline to a concentration of 4 nmol/μl. All ODNs were phosphorothioated on the three terminal bases of both 5′ and 3′ ends. This modification results in increased stability and less toxicity of the ODN. Sequences are as follows: BDNF-ASO, 5′-TCTTCCCCTTTTAATGGT-3′; BDNF-MSO, 5′-ATACTTTCTGTTCTTGCC-3′; Arc-ASO, 5′-GTCCAGCTCCATCTGCTCGC-3′; Arc-MSO, 5′-CGTGCACCTCTCGCAGCTTC-3′. All ODN sequences were subjected to a BLAST search on the National Center for Biotechnology Information BLAST server using the GenBank database. Control MSO sequence, which included the same 18 nucleotides as the ASO but in a scrambled order, did not generate any full matches to identified gene sequences in the database. Bilateral infusions were conducted simultaneously using two 5-μl Hamilton syringes that were connected to the infusion cannulas by propylene tubing. Syringes were driven by a Harvard Apparatus precision syringe pump, which delivered 0.5 μl to each hemisphere over 2 min. The infusion cannulas were left in place for an additional minute to allow for diffusion. At least 3 d were allowed for washout between repeated infusions.

### Immunoblot assays

After rats were killed, brains were immediately frozen and the Prh was microdissected. Tissue was homogenized in ice-chilled buffer (20 mM Tris-HCl [pH 7.4], 0.32 M sucrose, 1 mM EDTA, 1 mM EGTA, 1 mM PMSF, 10 mg/ml aprotinin, 15 mg/ml leupeptin, 10 mg/ml bacitracin, 10 mg/ml pepstatin, 15 mg/ml trypsin inhibitor, 50 mM NaF, and 1 mM sodium orthovanadate). Samples of homogenates (15 μg of protein) were subjected to 10% or 12% SDS-PAGE under reducing conditions. Proteins were transferred onto nitrocellulose membranes (Bio-Rad) in transfer buffer (25 mM Tris, 192 mM glycine, 10% vol/vol methanol) for 2 h at 100V. Western blots were performed by incubating membranes first with anti-BDNF antibody (N20, 1:1000, Santa Cruz Biotechnology), with anti-Arc antibody (1:2000, Santa Cruz Biotechnology) and anti-actin antibody (1:5000, Santa Cruz Biotechnology). One nanogram of recombinant human BDNF was used as a standard for Western blot (rhBDNF, Alomone). Blots were developed using enhanced chemiluminescence (GE Healthcare), visualized by Storm 845 PhosphorImager (GE Healthcare Life Sciences), and quantified using ImageJ software (NIH). For analysis, optical density (OD) values and the band areas were obtained for each microdissected hippocampal sample for both the target protein (BDNF, Arc) and the actin loading control. Each target OD value was normalized to its corresponding actin OD value, and normalized levels were averaged for each condition. Data were analyzed using a one-way ANOVA followed by Newman–Keuls *post hoc* comparisons. Data depicted in Fig. 2*D* were transformed before analysis.

### Apparatus

**Figure 1.**
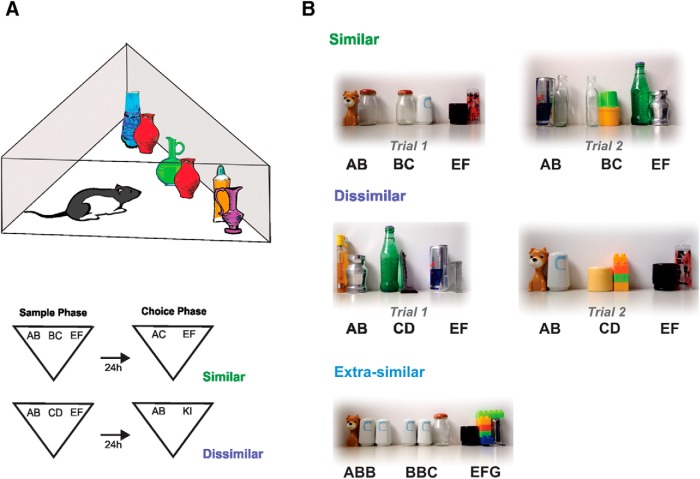
***A***, Left, cartoon depicting the apparatus and the spontaneous object recognition task (SOR). ***B***, Representative objects for trials 1 and 2 for the similar and dissimilar versions of the SOR task and the extra-similar version of the SOR task.

The triangular open field used for the spontaneous object recognition task (SOR) was made of white foam board. Each wall was 60 cm long by 60 cm high. The circular open field (90 cm diameter, 45 cm high) used for the spontaneous location recognition task (SLR) was made of black plastic. Both open fields were situated in the middle of a dimly lit room. The walls of the triangular open field were higher to minimize the visual access to the distal cues in the room. The circular open field was surrounded by four spatial cues and standard furniture. The open field floor was always covered with wood shavings. A video camera was positioned over the arena, and sample and choice phases were recorded for later analysis. The objects for the SOR task were made of two different smaller objects, except for the extra-similar condition, in which they were made by three smaller objects. Composite objects were made by simply attaching together two or three of the smaller items in the conditions described in Results (Fig. 1). We always used different objects for our within-subject design (examples can be seen in Fig. 1). For the SLR, the objects used were either soda cans or beer bottles from which the label had been removed. All objects were fixed to the floor of the open field with Blu-tack and cleaned with a 50% ethanol solution between sample and choice trials. For the SOR task, all three composite objects were aligned close to one of the walls of the arena, and positions within this line were pseudorandomly assigned. Other tasks that evaluate object discrimination have used objects built with LEGO. While LEGO-constructed objects offer some versatility when trying to manipulate the similarity between them, they could also cause more interference, as the texture would be the same between the different objects made of the same material. In fact, it has been shown that merely the fact that an object is built with LEGO can cause interference with another LEGO object that is not particularly similar (Bartko et al. 2010). Junk object features offer different textures and curvy shapes that are not present in LEGO-based objects.

For the SLR task (Fig. 5*D*,*E*), positions varied according to the condition tested, with objects always placed along a circumference 15 cm away from the wall and 30 cm away from the center of the arena. For the similar condition, objects were separated by a 50° angle; and for the dissimilar condition, they separated by an angle of 120°.

### Behavioral procedures

**Figure 2.**
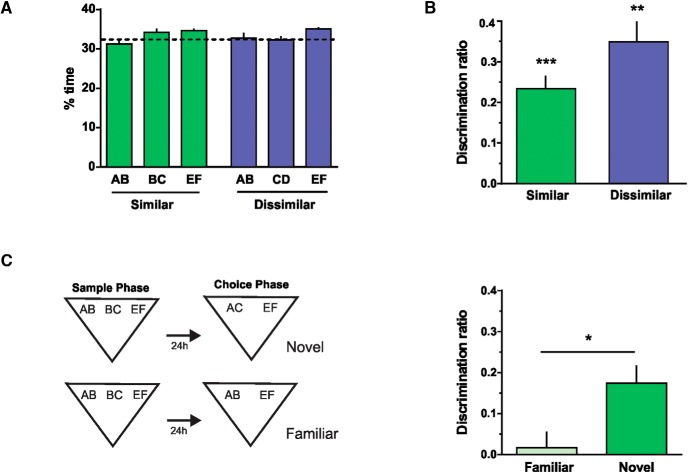
The spontaneous object recognition task. ***A***, Percentage of time spent exploring each of the objects in the sample phase in the dissimilar (left) and similar (right) condition. Rats spent an equal amount of time exploring each of the three objects during the sample phase. Similar: repeated-measures one-way ANOVA (%time), *F*_obj_ = 2.829, *p* = 0.125, *F*_ind_ = 1.624 × 10^–13^, *p* > 0.999; dissimilar: repeated-measures one-way ANOVA (%time), *F*_obj_ = 1.456, *p* = 0.274, *F*_ind_ = 1.014 × 10^–13^, *p* > 0.999. ***B***, Discrimination ratios during the choice phase, 24 h after the sample phase, in the similar and dissimilar condition. One-sample *t* test (similar, *t* = 8.11), *p* < 0.0001; one sample *t* test (dissimilar, *t* = 4.361), *p* = 0.003; similar versus dissimilar paired *t* test (*t* = 1.521), *p* = 0.172, *n* = 8. ***C***, Left, control task. Right, discrimination ratios during the choice phase for the novel and familiar conditions. Paired *t* test (*t* = 2.861), *p* = 0.0187, *n* = 10, *d* = 0.054. Data are expressed as the mean ± SEM; *, *p* < 0.05; **, *p* < 0.01; ***, *p* < 0.001.

**Figure 3.**
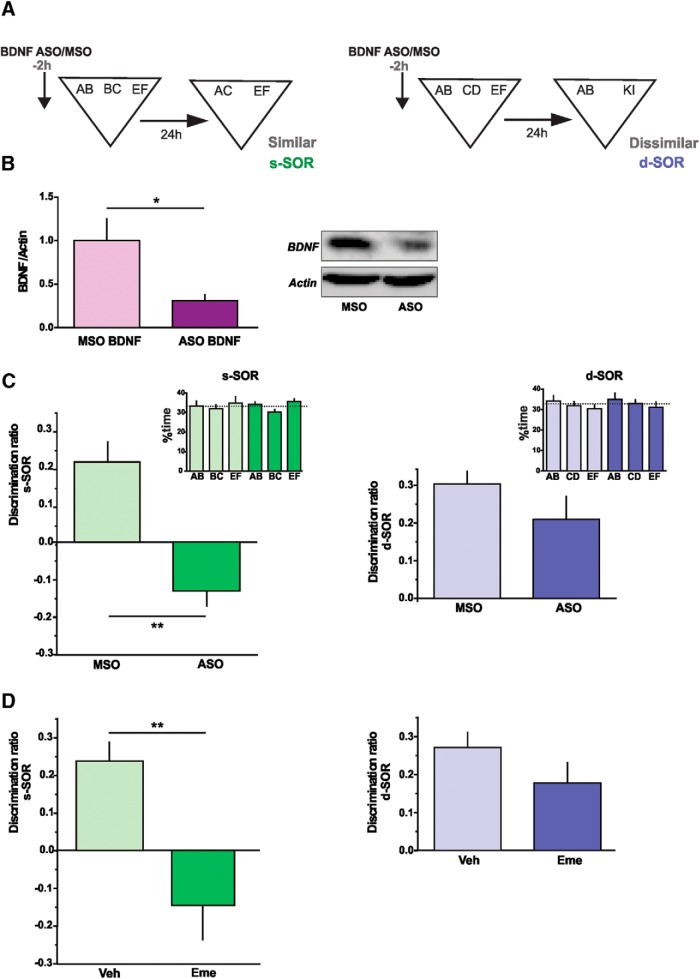
BDNF expression and protein synthesis in the Prh are required for consolidation of similar, but not dissimilar, object memory representations. ***A***, Schematic illustration of the two configurations of the SOR task depicting the time point at which BDNF-ASO was infused. ***B***, BDNF protein levels in the Prh of nontrained animals infused with either BDNF-ASO or BDNF-MSO 2 h before injection of kainic acid into the Prh. Unpaired *t* test (*t* = 2.334), *p* = 0.0322, *n* = 9–10, *d* = 0.377. ***C***, Effect of BDNF-ASO or BDNF-MSO injections on the discrimination ratios for the similar (s-SOR) and the dissimilar (d-SOR) version of the task. Paired *t* test (*t* = 4.284), *p* = 0.0036, *n* = 8–13, *d* = 2.284. Inset, percentage of time spent exploring each of the objects in the sample phase in the s-SOR (left) and d-SOR (right), 2 h after BDNF-MSO (light color) or BDNF-MSO (dark color). Similar: repeated-measures two-way ANOVA; *F* = 0.652, *p*(drug) = 0.440, *F* = 0.957, *p*(object) = 0.403, *F* = 0.135, *p*(interaction) = 0.875. Dissimilar: repeated-measures two-way ANOVA; *F* = 0.055, *p*(drug) = 0.818, *F* = 1.388, *p*(object) = 0.269, *F* = 0.001, *p*(interaction) = 0.999. ***D***, The injection of emetine in the PRH 15 min before the sample phase impaired performance on the s-SOR task during the choice phase 24 h later relative to vehicle-injected rats (left), whereas there was no effect of emetine on the d-SOR version of the task (right). Paired *t* test (s-SOR, *t* = 3.540), *p* = 0.0076, *n* = 9, *d* = 1.698; paired *t* test (d-SOR, *t* = 1.284), *p* = 0.231, *n* = 10. Data are expressed as the mean ± SEM; *, *p* < 0.05; **, *p* < 0.01.

For the SOR task (Fig. 1), each rat was handled for 3 d and then habituated to the arena for 5 min/d for 3 d before exposure to the objects (Figs. 2, 3, 5, 6, and 8). For the SLR task (Fig. 5*D*,*E*), each rat was handled for 3 d and then habituated to the arena for 10 min/d for 5 d before exposure to the objects. For the SOR task, after habituation, the rats were exposed during a 5-min duration sample phase to three objects made of either two or three features depending on the condition. For the similar condition, two of the objects shared one feature (AB and BC) and the third object was made of two other different features (EF). For the dissimilar condition, all three objects were made of different features (AB, CD, and EF). For the extra-similar condition (Fig. 8*A–D*), two of the objects shared two of three features (ABB and BBC), and the third one was different (EFG). The choice phase lasted 3 min and was conducted 24 h after the finalization of the sample phase. In this case, the animals were exposed to two objects, one novel and one familiar, that varied in composition according to the condition evaluated. For the similar condition, the novel object was made of the two nonshared features of the objects presented in the sample phase (AC), and the familiar object was a copy of the third object (EF). For the dissimilar condition, the novel object was made of two novel features (GH), and the familiar object was a copy of one of the objects presented during the sample phase (AB, CD, or EF). Because most of the experiments involved a within-subject design, the letters do not indicate that we used the same object or feature. We always used different objects and features for the different trials. The rationale behind the task was that if the rats were able to separate the two similar objects, their representations should be distinct and resistant to confusion; therefore, the rats should show preference for the novel object during the retrieval phase. However, if the representations of the two similar objects were not sufficiently separated, presentation of the new object would activate a familiar representation in memory and would thus not be distinguishable. The result would be that rats should behave as if the new object was familiar. As this process is thought to happen during encoding/consolidation stages of memory formation, the similarity of the to-be-remembered objects was varied during encoding/consolidation, rather than the retrieval phase of the task. Unlike other tests of discrimination (Gilbert et al. 1998; Clelland et al. 2009; Nakashiba et al. 2012), the use of a continuous variable as a measure of performance yields sufficient data within a single trial to allow manipulations at different stages of memory. In contrast, previous tasks using discrete trial procedures required many trials to collect sufficient data, and thus such manipulations would have to be repeated an impracticable number of times.

For the extra-similar condition (Fig. 8*A–D*), the novel object was made of a novel combination of familiar features (ABC), and the familiar object was a copy of the third object presented in the sample phase (EFG). Exploration was recorded and later scored manually for both the sample and choice phases. For all experiments, exploration of a particular object was defined as the rat having its nose directed at the object at a distance of 2 cm or less, or touching the object with its nose. Rearing with the head oriented upward did not count as exploration. Climbing over or sitting on the objects was not included. Two people scored the videos; one was blind to the novel and familiar objects. There was no significant interrater variability.

For the SLR task (Fig. 5*D*,*E*), after habituation, rats were exposed to three identical objects, A1, A2, and A3, during a sample phase that lasted for 10 min. For the similar SLR (s-SLR), objects A2 and A3 were placed 50° apart (20.5 cm between them) and object A3 at an equal distance from the other two. For the dissimilar SLR (D-SLR), objects A1, A2, and A3 were equidistant, 120° (49 cm between them) apart from each other. Twenty-four hours after the sample phase, rats were exposed to two new identical copies of the objects, A4 and A5, for 5 min. New identical copies were used to prevent the use of olfactory cues. During this choice phase, object A4 was placed in a familiar location (same position as in the sample phase) and object A5 was placed in a novel location. For the s-SLR task, the novel location was defined as a position exactly in between the ones in which objects A2 and A3 were located during the sample phase (see schemes in Fig. 5*D*). For the D-SLR task, object A4 was placed in a familiar location and object A5 in a position equidistant to the previous locations of A2 and A3 (see schemes in Fig. 5*D*).

### Experimental design and statistical analysis

**Figure 4.**
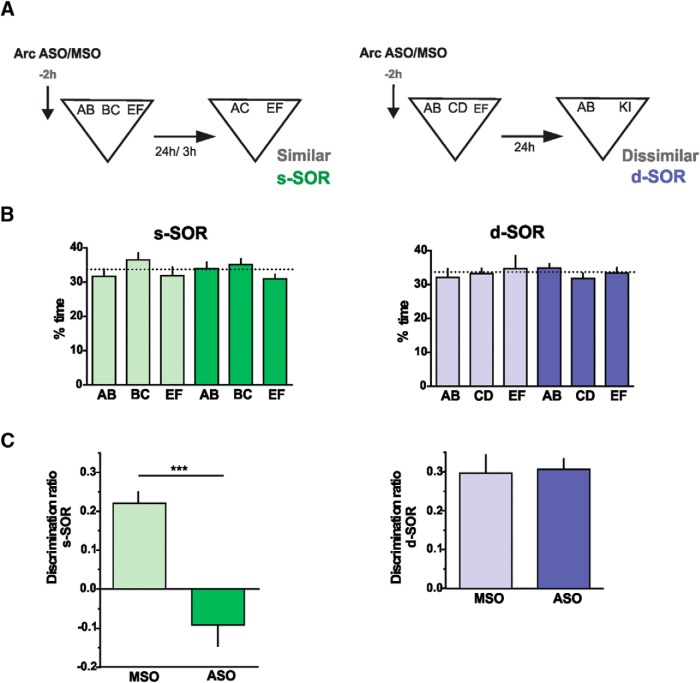
***A***, Arc expression in the Prh is required for consolidation of similar, but not dissimilar, object memory representations. ***B***, Percentage of time spent exploring each of the objects in the sample phase, 2 h after MSO (light color) or ASO (dark color) of Arc injection. Similar: repeated-measures two-way ANOVA; *F* = 0.026, *p*(drug) = 0.875, *F* = 1.561, *p*(object) = 0.240, *F* = 0.256, *p*(interaction) = 0.777. Dissimilar: repeated-measures two-way ANOVA; *F* = 4615, *p*(drug) = 0.522, *F* = 0.1971, *p*(object) = 0.824, *F* = 0.2516, *p*(interaction) = 0.782. (C) Effect of presample injection of Arc-ASO or Arc-MSO into the Prh in the choice phase at 24 h in the s-SOR (left) or the d-SOR (right) version of the task. Paired *t* test (s-SOR, *t* = 5.762), *p* = 0.0002, *n* = 11, *d* = 7599; paired *t* test (d-SOR, *t* = 0.421), *p* = 0.683, *n* = 11. Data are expressed as the mean ± SEM; ***, *p* < 0.001.

**Figure 5.**
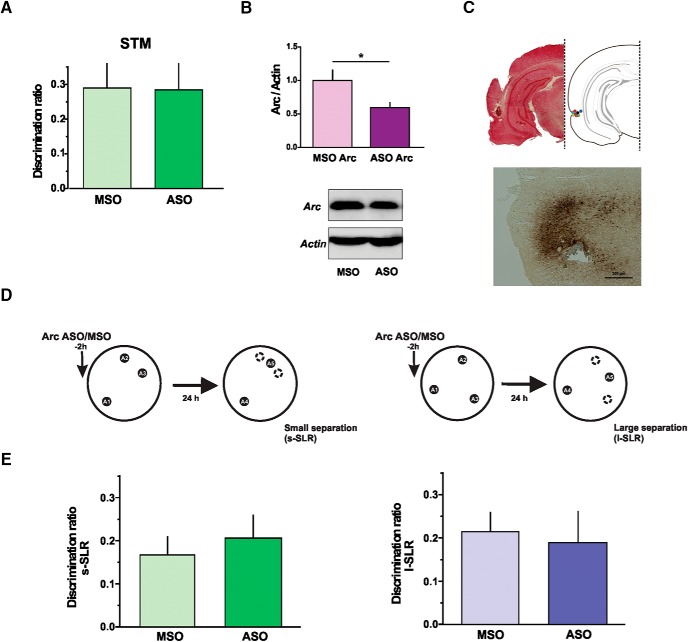
Arc expression in the Prh is not necessary for discrimination of overlapping spatial representations or for short-term memory. ***A***, Short-term memory test after the injection of Arc-ASO or MSO 2 h previous to the s-SOR. Paired *t* test *p* = 0.974, *t* = 0.0343, *n* = 6. Data are expressed as the mean ± SEM. ***B***, Arc protein levels in the Prh of nontrained animals infused with either Arc-ASO or MSO 2 h before injection of kainic acid into the Prh. Unpaired *t* test *p* = 0.046, *t* = 2.317, *n* = 5–6, *d* = 1.644. ***C***, Upper panel, coronal section showing the track of the cannula and indicating representative infusion sites in the Prh. Lower panel, representative spread of a biotinylated Arc-ASO in the Prh 2 h after injection of 2 nmol. ***D***, Schematic representation of the similar configuration (s-SLR, left) and dissimilar configuration of the spontaneous location recognition task (L-SLR, right) showing the time of infusion of Arc-ASO or MSO. ***E***, Effect of Arc-ASO or Arc-MSO infusion into Prh in the SLR task. Paired *t* test (s-SLR, *t* = 0.521), *p* = 0.618; paired *t* test (D-SLR, *t* = 0.713), *p* = 0.499, *n* = 8. Data expressed as the mean ± SEM; *, *p* < 0.05.

**Figure 6.**
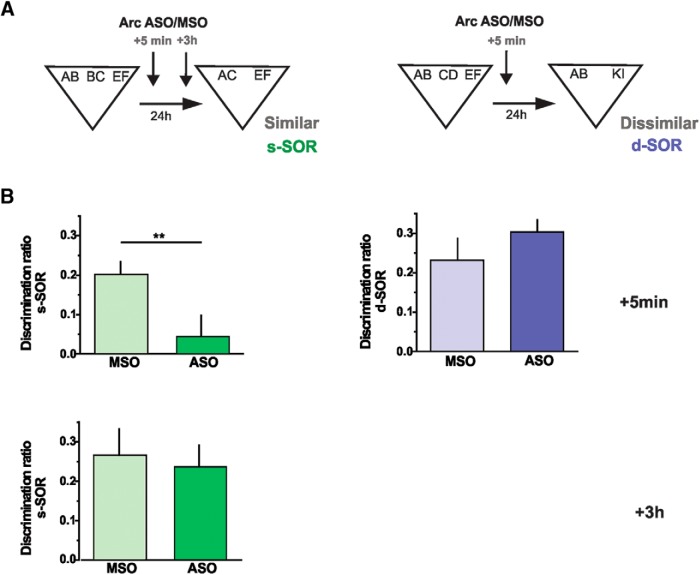
Arc expression in the Prh is required in a time-restricted window for consolidation of similar object memory representations. ***A***, Schematic illustration of the similar (s-SOR, left) or dissimilar (d-SOR right) task configurations depicting the time points at which Arc-MSO or ASO was infused. ***B***, Effect of the injection of Arc-ASO or Arc-MSO into the Prh 5 min or 3 h after the sample phase in the s-SOR (left) or the d-SOR (right) version of the task evaluated in a choice phase 24 h later. Paired *t* test (similar 0 h, *t* = 2.274), *p* = 0.046, *n* = 11, *d* = 1.611; paired *t* test (dissimilar 0 h, *t* = 0.999), *p* = 0.351, *n* = 8; paired *t* test (similar 3 h, *t* = 0.459), *p* = 0.663, *n* = 7. Data are expressed as the mean ± SEM; **, *p* < 0.01.

For all the experiments, the results were expressed as a discrimination ratio that was calculated as the time exploring the novel object (SOR) or the object in the novel location (SLR) minus the time exploring the familiar object (SOR) or the object in the familiar location (SLR) divided by total exploration time [(*t*_novel_ – *t*_familiar_)/*t*_total_]. In Fig. 2*C*, one-sample *t* test was used to compare the discrimination ratio from the similar and dissimilar conditions to verify that the ratio was different from zero. For the experiment shown in Fig. 2*C*, half of the rats were tested first in the “novel condition” and then in the “familiar condition,” and the other half were tested first for the familiar and then for the novel conditions. Discrimination ratios were compared within subject using a paired *t* test. For experiments shown in Figs. 3*C*,*D*, 4*C*, 5*A*,*E*, and 6*B*, rats were tested twice. In the first trial, half of the animals received ASO injection and the other half received MSO injection. In the second trial, they were injected with either ASO or MSO depending on what they had received in the first trial. For the sample phase, the percentage of time exploring each object was compared using a repeated-measures two-way ANOVA, with time and object as the repeated measures. For the choice phase, discrimination ratios were compared within subject using a paired *t* test. Different features (A, B, C, etc.) were used to reproduce the same task conditions in the consecutive trials of the within-subject design. For the experiment in Fig. 8*F*, animals were tested twice, once injected with Arc-ASO and BDNF-ASO in the hemisphere and once with Arc-ASO and BDNF-ASO in different hemispheres. Control MSO was injected in the other hemisphere. Discrimination ratios were compared within subject using a paired *t* test. For the experiments shown in Fig. 8*B*,*D*, animals were tested only once, and discrimination ratios were analyzed using a *t* test, or a two-way ANOVA followed by Bonferroni *post hoc* comparisons. In all experiments, drug and vehicle or ASO and MSO injections were counterbalanced. We performed one-sample *t* tests for every discrimination ratio to analyze whether control animals learned the task.

### Histology

At the completion of behavioral testing, all rats except the ones used for additional experiments were anaesthetized by i.p. injection with 2 ml of Euthatal (Rhône Merieux) and perfused transcardially with PBS, followed by 10% neutral buffered formalin. The brains were removed and postfixed in formalin for at least 24 h before being immersed in 20% sucrose solution until they sank. Sixty-micrometer sections were cut on a freezing microtome encompassing the extent of the injector track. Every fifth section was mounted on a gelatin-coated glass slide and stained with cresyl violet. Slides were examined under a light microscope to verify the location of the injections. For analysis of ODN spread after injection, rats were injected with 2 nmol/μl (0.5 μl/side) of biotinylated Arc-ASO ODN, and 2 h later, they were anesthetized and perfused transcardially with 0.9% saline followed by 4% paraformaldehyde. The brains were isolated and sliced, and the ASO was detected by avidin–biotin staining (Bekinschtein et al. 2007)

## Results

In the original SOR task (Ennaceur and Delacour, 1988; Warburton et al. 2000), rats are exposed during a sample phase to two identical objects placed within an arena. After a variable delay, rats are given a choice phase in which one of the objects is replaced by a completely novel object. Because rats naturally prefer novelty, rats with intact memory spend significantly more time exploring the novel object than the familiar one (Warburton et al. 2000). A detailed description of the modified task we used in this study can be found in Methods. Briefly, it consisted of a sample (study) phase in which rats are exposed to three objects, two of them similar to each other (AB and BC) and the third object dissimilar (EF; Fig. 1). This task is analogous to our SLR, which was developed as a test for spatial discrimination of overlapping memories (Bekinschtein et al. 2013). In SLR, the similarity between the spatial representations was manipulated by varying the distance between identical objects. In the analogous task used in the present study to evaluate discrimination of overlapping object memories during consolidation, the similarity between objects was manipulated by varying the number of features shared by them at the encoding phase (Fig. 1).

### Object exploration and preference is driven by novelty in the modified SOR task

There were no differences in the percentage of time the animals spent exploring the three objects during the sample phase for the similar or the dissimilar conditions (Fig. 2*A*). In addition, the total amount of time rats spent exploring did not differ between conditions (similar versus dissimilar: paired *t* test, *p =* 0.943). The choice phase or test was conducted 24 h after the sample phase, and memory was evaluated by comparing the amount of time spent exploring a novel object and a familiar object. In the similar condition, the novel object was made of the nonoverlapping (AC) features of the two similar objects from the sample phase (AB and BC), and the familiar object was a copy of the third one presented in the sample phase (EF; Fig. 1). Rats spent significantly more time exploring the novel than the familiar object (Fig. 2*B*, Table 3), indicating that they were able to store separate representations of the similar objects presented during the sample phase and to recognize the new object as novel despite it being made of familiar features. A similar result was obtained for the dissimilar condition in which a novel object made of two completely new features (KL) was paired against a familiar object seen during the sample phase (AB, CD, or EF; Fig. 2*B*).

These results indicate that intact animals were able to spontaneously disambiguate the representations of two similar objects seen 24 h before the test. However, there was a possibility that the rats explored the novel object more during the choice phase due to a change in the number of items from three to two between the sample and the choice phases. To rule out that the difference in the novelty coming from the change in the number of objects was driving exploration preferentially to one of them, we presented two familiar objects during the choice phase and compared either AB or BC against EF (Fig. 2*C*). There was no preference for any of the two objects after this manipulation, indicating that item novelty was the main driver for exploration in this task (Fig. 2*C*, Table 3). Although in the novel condition, the discrimination ratio was different from zero, this was not the case for the familiar condition (*p*_fam_ = 0.68, *t* = 0.43; *p*_novel_ = 0.016, *t* = 3.97; one-sample *t* test). Object location was always pseudorandomly assigned in case there was a bias for location within the arena.

### BDNF and protein synthesis are required for the discrimination of overlapping object representations in Prh

Long-term storage of information in the brain is thought to require structural changes at the synapses (Kandel, 2001). Stable forms of synaptic plasticity and memory have long been known to depend on neuronal activity-induced protein synthesis (Davis and Squire, 1984; McGaugh, 2000). BDNF is a neurotrophin shown to be essential for memory consolidation in different learning tasks, including object recognition (Bekinschtein et al. 2014a). In addition, BDNF can induce long-term potentiation in the DG (Messaoudi et al. 2007). We have previously demonstrated that BDNF is required for consolidation of overlapping spatial memories in the DG (Bekinschtein et al. 2013); thus, we hypothesized that it may participate in this process in Prh as well.

To evaluate the requirement of BDNF in the SOR task, we injected an antisense oligonucleotide for BDNF (BDNF-ASO) or a missense control oligonucleotide with the same base composition but in a random order (BDNF-MSO) in Prh 2 h before the sample phase for the similar and dissimilar versions of the SOR task (Fig. 3*A*). To first ensure that BDNF-ASO was efficiently blocking BDNF expression in Prh, we infused either ASO or MSO 2 h before injection of kainic acid or vehicle into the Prh of naive animals. This method was previously used to induce immediate-early genes (Nakayama et al. 2015). Thirty minutes after kainic acid injection, the Prh was dissected out and processed for Western blot analysis of BDNF protein content. BDNF-ASO, but not BDNF-MSO, was able to block the increase in BDNF expression caused by kainic acid (Fig. 3*B*), indicating that the ASO was effectively preventing BDNF expression. It is unlikely that BDNF-ASO reduced steady-state levels at the time of the sample phase. Previous experiments using fear learning have shown an amnesic effect on long-term memory of presample BDNF blocking antibodies, but not of BDNF-ASO, suggesting that BDNF-ASO acts only on *de novo* BDNF synthesis (Slipczuk et al. 2009). Although in this work we did not perform a dose–response curve of BDNF-ASO on BDNF protein levels, previous work showed that 2 h postinjection, there were no differences in BDNF steady-state levels between BDNF-ASO and BDNF-MSO in the dorsal hippocampus (Bekinschtein et al. 2007). This also suggests that in these experiments, BDNF-ASO blocks BDNF expression induced by learning. Animals in both groups explored the three objects equally (Fig. 3*C*, inset; Table 1). When the animals were evaluated 24 h later, we found a significant difference in the discrimination ratio between BDNF-ASO– and BDNF-MSO– injected animals only for the similar SOR (Fig. 3*C*), but no differences in total exploration times (see Table 5; paired *t* test, *p*_similar_ = 0.945, *p*_dissimilar_ = 0.523,). One-sample *t* test indicate that BDNF-MSO–injected animals did learn the s-SOR and d-SOR tasks (*p*_similar_ = 0.01, *t* = 3.38; *p*_dissimilar_ < 0.0001, *t* = 8.55), whereas BDNF-ASO–injected animals learned only the d-SOR task (*p*_similar_ = 0.16, *t* = 3.14; *p*_dissimilar_ = 0.006, *t* = 3.35). We have seen negative discrimination ratios before, but see Discussion for an interpretation of this particular result. This indicates that BDNF is required for acquisition and/or consolidation of overlapping object memories in Prh.

**Table 1.** Total exploration times during the sample sessions for Figs. 2, 3, and 4

Figure	AB	BC/CD	EF
2*A*			
s-SOR	32.40 ± 1.72	37.75 ± 2.98	36.22 ± 2.25
d-SOR	34.23 ± 2.45	33.79 ± 2.12	38.42 ± 2.91
3*C*			
s-SOR			
BDNF-MSO	27.76 ± 4.06	27.59 ± 4.00	34.14 ± 4.09
BDNF-ASO	31.06 ± 4.83	27.54 ± 5.30	32.04 ± 5.41
d-SOR			
BDNF-MSO	26.07 ± 3.06	24.64 ± 3.70	23.26 ± 3.77
BDNF-ASO	24.93 ± 2.94	27.75 ± 3.72	25.65 ± 3.12
3*D*			
s-SOR			
Vehicle	31.82 ± 2.99	25.98 ± 2.92	28.81 ± 3.27
Emetine	30.86 ± 5.35	25.21 ± 3.91	29.84 ± 4.10
d-SOR			
Vehicle	32.58 ± 3.70	30.92 ± 3.94	31.38 ± 3.28
Emetine	34.37 ± 4.05	31.98 ± 5.09	30.48 ± 3.92
4*B*			
s-SOR			
Arc-MSO	36.06 ± 3.07	43.03 ± 2.87	38.44 ± 3.54
Arc-ASO	39.24 ± 3.36	39.27 ± 3.82	36.21 ± 4.51
d-SOR			
Arc-MSO	38.74 ± 2.63	38.19 ± 1.66	42.46 ± 4.46
Arc-ASO	37.39 ± 2.48	33.49 ± 2.81	36.15 ± 3.51

Results are expressed as mean ± SEM in seconds.

If BDNF was specifically involved in consolidation, then infusion of the BDNF-ASO should not affect short-term memory. To evaluate this, we injected BDNF-ASO or MSO into Prh and tested short-term memory in the similar version of the task. We did not find a significant difference between ASO and MSO. Both ODNs were infused 2 h before the sample phase, and memory was evaluated 3 h postacquisition. We found that both groups remembered equally (BDNF-MSO DR 0.23 ± 0.03 versus BDNF-ASO DR 0.24 ± 0.03, *n* = 7, *p* = 0.63, *t*_6_ = 0.50, paired *t* test). We next asked whether specific expression of BDNF was involved in the process of consolidating overlapping memories and whether other molecules could participate in a process of storing nonoverlapping memories in Prh. If this were the case, contrary to the effects of BDNF blockade, general inhibition of protein synthesis in Prh should impair SOR both in the similar and the dissimilar condition. To block protein synthesis, we injected the translation inhibitor emetine (Sigma-Aldrich) into Prh, 15 min before the sample phase in both the similar and dissimilar conditions. When memory was evaluated 24 h later, we found a deficit for the emetine-injected group only in the similar condition (Fig. 3*D*, left). No memory impairment was observed in emetine-injected animals that were evaluated in the dissimilar condition (Fig. 3*D*, right). One-sample *t* tests indicated that vehicle-injected animals were able to learn both the s-SOR and the d-SOR (*p*_similar_ = 0.001, *t* = 4.75; *p*_dissimilar_ < 0.0001, *t* = 6.67), whereas emetine-injected animals learned only the d-SOR version (*p*_similar_ = 0.16, *t* = 1.5; *p*_dissimilar_ = 0.01, *t* = 3.22). These results suggest that protein synthesis in Prh is required for consolidation of overlapping, but not nonoverlapping, memories and that BDNF participates in a general protein synthesis–dependent mechanism of disambiguation of object memories in Prh.

### Arc/Arg3.1 expression is required for the discrimination of overlapping object memories in Prh

We then decided to look for a potential effector of BDNF in Prh. Most studies have focused on the study of Arc in brain regions such as the hippocampus and amygdala, and there is no information regarding the role of Arc in object recognition in Prh or specifically in pattern separation. In addition, BDNF-induced long-term potentiation in the DG is dependent on Arc synthesis (Messaoudi et al. 2007). Thus we hypothesized that Arc expression could be induced by BDNF in Prh during consolidation of similar object memories.

We focused this set of experiments on the function of the Arc protein in Prh during storage and disambiguation of object representations. As with BDNF, the expression of Arc can be efficiently blocked by the application of antisense oligonucleotides (ASO) that bind specifically to the Arc mRNA (Messaoudi et al. 2007; Ploski et al. 2008; Martínez et al. 2012; Nakayama et al. 2015). We infused Arc-ASO or a control missense oligonucleotide (Arc-MSO) in Prh 2 h before the sample phase and tested the animals 24 h later. Infusion of the ODNs did not affect total exploration times during the sample phase (see Table 5; ASO versus MSO, paired *t* test, *p*_similar_ = 0.585; *p*_dissimilar_ = 0.919), and rats spent an equal amount of time exploring each one of the three objects (Fig. 4*B*, Table 1). However, infusions of the ODNs impaired object recognition memory for the similar, but not for the dissimilar, condition (Fig. 4*C*). One-sample *t* tests indicate that Arc-MSO–injected animals were able to learn both the s-SOR and the d-SOR (*p*_similar_ < 0.0001, *t* = 7.14; *p*_dissimilar_ < 0.0001, *t* = 11.8), whereas Arc-ASO–injected animals learned only the d-SOR version (*p*_similar_ = 0.13, *t* = 1.64; *p*_dissimilar_ < 0.0001, *t* = 10.8). No memory impairment was observed when Arc-ASO was infused 2 h before the sample phase and the animals were evaluated after 3 h (Fig. 5*A*). One-sample *t* tests indicated that both Arc-MSO– and Arc-ASO–injected animals were able to remember the s-SOR task at 3 h (*p*_similarMSO_ = 0.04, *t* = 2.8; *p*_similarASO_ = 0.02, *t* = 3.3). There were no differences in total exploration times between ASO- and MSO-injected animals during the choice phase (see Table 4; paired *t* test, *p*_similar_ = 0.206; *p*_dissimilar_ = 0.875). This indicates that initial acquisition of the task was not affected by Arc blockade and that the effect of this manipulation was dependent on the delay between sample and choice, suggesting that the effect was happening during the consolidation phase. To ensure that Arc-ASO was efficiently blocking Arc expression in Prh, we infused either ASO or MSO 2 h before injection of kainic acid or vehicle into the Prh of naive animals. Thirty minutes after kainic acid injection, the Prh was dissected out and processed for Western blot analysis of Arc protein content. Arc-ASO, but not Arc-MSO, was able to block the increase in Arc expression caused by kainic acid (Fig. 5*B*), indicating that the ODN was effectively preventing Arc expression.

These results cannot be explained by unspecific damage to Prh by the oligonucleotide Arc-ASO, because no change in performance was seen after administering Arc-MSO, and staining did not reveal any lesion to the site of infusion (Fig. 5*C*). In addition, the experimental design was within-subject, so every rat was both injected with ASO and MSO. Thus, it is very unlikely that ASO and MSO had differential toxic effects that were somehow reversible. We evaluated ODN spread 2 h after injection of biotinylated Arc-ASO into Prh. We found little spread outside Prh, indicating that the observed deficit was not caused by blocking Arc expression in other structures (Fig. 5*C*).

### Arc expression in Prh is not necessary for DG-dependent discrimination of overlapping spatial representations

Another interpretation of these results could be that Arc is required in Prh for discrimination of similar information of any kind or that the impairment is evident or not depending on the difficulty of the task. If this were the case, then disambiguation of similar information, regardless of the type of stimuli involved, should also be affected by injection of Arc-ASO into Prh. To evaluate this possibility, we tested the rats in a spontaneous spatial discrimination task that is particularly sensitive to manipulations of the DG (Bekinschtein et al. 2013, 2014b; Fig. 5*D*). As with our version of the SOR, the spontaneous location recognition task (SLR) can be run in two different conditions, the similar/small separation (s-SLR) and the dissimilar/large separation (l-SLR) configurations (Fig. 5*D*). Similarity of the locations can be manipulated by varying the distance between the objects within a circular arena surrounded by distal spatial cues. The s-SLR, but not the l-SLR is sensitive to DG manipulations like blockade of BDNF (Bekinschtein et al. 2013) or adult neurogenesis (Bekinschtein et al. 2014b; Reichelt et al. 2016). Infusion of Arc-ASO in Prh 2 h before the sample phase did not produce any observable deficit in the SLR task for any of the conditions (Fig. 5*E*, Table 3). One-sample *t* tests indicate that both Arc-MSO– and Arc-ASO–injected animals were able to learn the s-SLR and l-SLR task (*p*_similarMSO_ = 0.006, *t* = 3.86; *p*_similarASO_ = 0.007, *t* = 3.76; *p_dis_*_similarMSO_ = 0.002, *t* = 4.73; *p_dis_*_similarASO_ = 0.04, *t* = 2.56). These results indicated that disambiguation of spatial overlapping information does not require Arc in Prh.

### Arc expression is necessary for discrimination of overlapping object memories in Prh during a time-restricted window

Memory consolidation is a time-restricted process, with amnestic agents being effective only during a limited time window (McGaugh, 2000; Winters and Bussey, 2005a). To test whether Arc requirement for LTM of the similar SOR was limited to the first few hours after the sample phase, Arc-ASO was injected into Prh either immediately or 3 h after the sample phase, and rats were tested 24 h after acquisition. We found a significant effect of Arc-ASO compared with Arc-MSO when the injection was made immediately after the sample phase, but only for the similar condition (Fig. 6*B*). One-sample *t* tests indicated that MSO-injected animals were able to learn both the s-SOR and the d-SOR (*p*_similar_ = 0.0001, *t* = 6.2; *p*_dissimilar_ = 0.0049, *t* = 4.04), whereas ASO-injected animals learned only the d-SOR version (*p*_similar_ = 0.43, *t* = 0.81; *p*_dissimilar_<0.0001, *t* = 9.1). We did not observe any memory impairment in the similar SOR when the Arc-ASO was injected in Prh 3 h after the sample phase (Fig. 6*B*, bottom), indicating that the effect of Arc-ASO was time-restricted. Injection of the Arc-ASO did not change total exploration times compared with Arc-MSO (see Table 5; paired *t* test, *p*_similar_ = 0.837; *p*_dissimilar_ = 0.654). In addition, one-sample *t* tests indicated that both Arc-MSO– and Arc-ASO–injected animals were able to learn the s-SOR (*p*_similarMSO_ = 0.009, *t* = 3.75; *p*_similarASO_ = 0.005, *t* = 4.26). The timing of infusion was conducted as previously described for this and other ODNs. The presample time was chosen because ODNs are slowly taken by cells, so for them to have an effect on *de novo* synthesis, they need to be injected at least 1.5 h before the experience. Thus, the ODNs injected 3 h postsample might affect protein synthesis at ∼4.5 h post-sample, when consolidation seems to have ended. These results are similar to the ones obtained when infusing Arc-ASO into the amygdala to block fear extinction (Onoue et al. 2014): pre-extinction infusion caused an impairment, but infusion 3 h after extinction training did not produce any effect.

### Arc expression in Prh increased as needed

The findings of these experiments provide compelling evidence that Arc in Prh is involved in the molecular mechanisms underlying the disambiguation of overlapping object memories. Moreover, these findings isolate the action of Arc to the consolidation phase of memory, specifically. Particularly interesting is the finding that postsample injections, made after initial encoding of the to-be-remembered objects, disrupt memory only in the similar SOR but not in the dissimilar SOR. This finding raises the question of whether Arc is expressed equally in both conditions but needed only in the first, or whether Arc is expressed on an “as-needed” basis, that is, spontaneously in response to encountering similar objects, the representations of which need to be separated before storage in memory. We have previously found that BDNF was expressed in this manner in the DG after exposure to similar locations (Bekinschtein et al. 2013).

**Figure 7.**
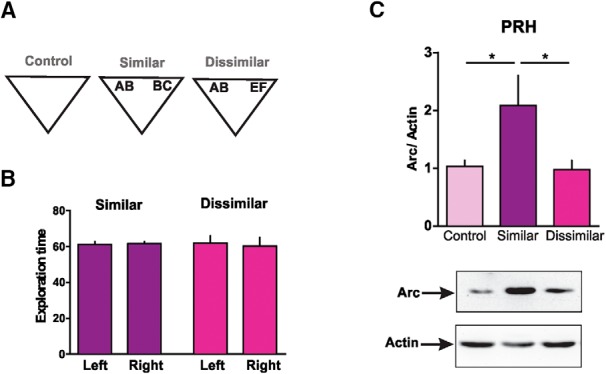
Exploration of similar objects, but not dissimilar objects, is associated with an increase in the protein levels of Arc in the Prh. ***A***, Schematic representations of the task configurations. ***B***, Percentage of exploration of the objects used during the similar and dissimilar task, considering the location (left or right) of the object during the task. ***C***, Arc protein levels in the Prh after exposure to the objects. One-way ANOVA, *F* = 3.818, *p* = 0.038, *n* = 8. Control versus similar: *d* = 2.407; Dissimilar versus similar: *d* = 2.073. Data are expressed as the mean ± SEM; *, *p* < 0.05.

To test this possibility, we exposed rats to two similar objects or two dissimilar objects within the training arena and a control group to the empty arena (Fig. 7*A*). One hour after the exposure, rats were killed, and the Prh was dissected and homogenized for Western blot analysis of Arc protein content. There were no differences in total exploration times, and rats spent an equal amount of time exploring each object in the similar and the dissimilar conditions (two-way ANOVA (%time) *p*_position_ = 0.943, *p*_condition_ = 0.673, *p*_interaction_ = 0.591; *t* test (total time) *p =* 0.943; Fig. 7*B*). Immunostaining revealed a one-fold increase in Arc expression in the animals exposed to the two similar objects compared with the ones exposed either to the two dissimilar objects or to the empty arena (Fig. 7*C*). These findings provide evidence that Arc is expressed on an as-needed basis, such that Arc is increased spontaneously when separating the representations of similar objects. Although we tried measuring BDNF, its levels proved difficult to measure because of its low expression in Prh. Nonetheless, BDNF-ASO caused amnesia only for the similar condition, indicating that synthesis of BDNF was required only to consolidate overlapping memories.

### BDNF enhances discrimination of overlapping object memories in Prh through Arc expression

**Figure 8.**
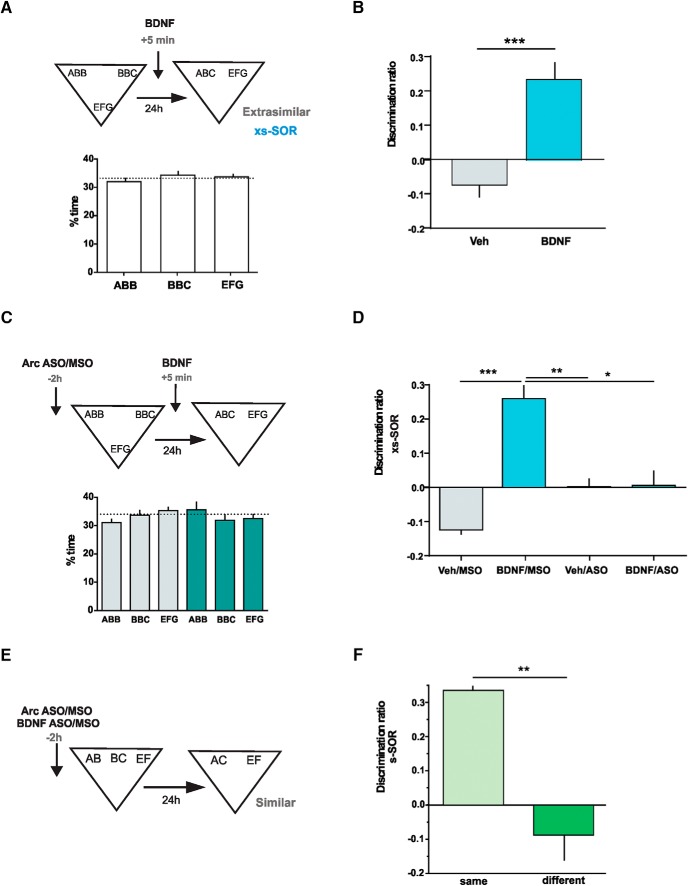
Arc and BDNF molecular pathways interact during consolidation of similar object representations in Prh. ***A***, Bottom, percentage of time spent exploring each of the objects in the sample phase in the xs-SOR. One-way ANOVA (%time), *F* = 0.845, *p* = 0.436. ***B***, Rats injected with recombinant BDNF in the Prh 5 min after the sample phase. Unpaired *t* test (*t* = 5.224), *p* = 0.0001, *n* = 8, *d* = 2.612. ***C***, Percentage of time spent exploring each of the objects in the sample phase in the xs-SOR after the injection of Arc-MSO (light color) or ASO (dark color). Two-way ANOVA (%time) *F* = 1.496, *p*(drug) = 0.235; *F* = 0.098, *p*(object) = 0.907; *F* = 1.358, *p*(interaction) = 0.269. ***D***, Effects of the combined injection of BDNF and Arc-ASO on the discrimination ratio in the xs-SOR. Two-way ANOVA *F* = 14.95, *p*(BDNF) = 0.001; *F* = 1.627, *p*(Arc-ASO) = 0.217; *F* = 14.29, *p*(interaction) = 0.0012; *n* = 6. BDNF/MSO versus BDNF/ASO: *d* = 1.796; Veh/MSO versus BDNF/MSO: *d* = 1.411; Veh/ASO versus BDNF/MSO: *d* = 0.294. ***E***, Schematic illustration of the s-SOR task and infusion time points. ***F***, Effects of the injection of an Arc-ASO and BDNF-ASO in the Prh of the same or opposite hemispheres on performance of the s-SOR task. Paired *t* test (*t* = 4.338), *p* = 0.0074, *n* = 6, *d* = 7.383. Data are expressed as the mean ± SEM; *, *p* < 0.05; **, *p* < 0.01; ***, *p* < 0.001.

We then asked whether BDNF and Arc expression in Prh during consolidation of overlapping memories were part of the same or different pathways. Because BDNF has been shown to enhance memory consolidation when injected exogenously (Alonso et al. 2002; Peters et al. 2010; Bekinschtein et al. 2013), we reasoned that this putative enhancing effect should be prevented if Arc expression was blocked. In addition, it has been shown previously that hrBDNF induces Arc expression in the hippocampus (Ying et al. 2002; Lee et al. 2004). To be able to see memory enhancement, we brought control animals’ performance down to chance levels by making the discrimination more difficult. Thus, we modified the task by making the objects more similar during the sample phase. For this extra-similar SOR (xs-SOR), we used objects made of three features; two of these objects shared two of the features (ABB and BBC), and the third object was completely different from the other two (EFG; Fig. 8*A*, see also Fig. 1). We evaluated memory 24 h after the sample phase using one novel object made of the repeated feature and the other two nonshared features (ABC) and a familiar object (EFG; Fig. 8*B*). There were no differences in exploration of the three objects during the sample phase, indicating that making two objects even more similar did not affect visual or tactile perception of them (Fig. 8*A*, bottom, Table 2). The discrimination ratio for control saline-injected rats was not significantly different from zero, indicating that they could not store the representations of the two similar objects as different (Fig. 8*B*, *p*_xsVeh_ = 0.08, *t* = 2.02, one-sample *t* test). However, injection of human recombinant BDNF (hrBDNF) into Prh 5 min after the sample phase enhanced performance compared with the control group (Fig. 8*B*, Table 3). In addition, a one-sample *t test* revealed that the discrimination ratio of BDNF-injected animals was significantly different from zero (*p*_xsBDNF_ = 0.0015, *t* = 5.06). This indicates that infusion of BDNF into Prh improved the consolidation of overlapping object memories.

**Table 2.** Total exploration times during the sample sessions for Fig. 8

Figure	ABB	BBC	EFG
8*A*	37.52 ± 8.03	37.56 ± 10.46	38.62 ± 9.59
8*C*			
Arc-MSO	36.75 ± 2.67	39.66 ± 3.14	41.47 ± 2.64
Arc-ASO	41.80 ± 2.39	39.02 ± 3.20	40.01 ± 3.37

**Table 3.** Total exploration times during the choice session of the SOR and SLR tasks

Fig. 2*B*	Novel	Familiar
Similar	24.9 ± 1.6	15.6 ± 1.3
Dissimilar	32.3 ± 2.6	15.4 ± 1.8
2*C*		
Familiar condition	28.4 ± 2.2	27.0 ± 3.2
Novel condition	32.5 ± 3.2	23.6 ± 1.6
3*D*,*E*		
s-SOR		
BDNF-MSO	30.5 ± 2.8	20.2 ± 2.4
BDNF-ASO	23.5 ± 4.7	28.3 ± 4.3
d-SOR		
BDNF-MSO	37.3 ± 3.1	20.2 ± 2.1
BDNF-ASO	38.3 ± 3.7	24.2 ± 2.7
3*F*,*G*		
s-SOR		
Vehicle	31.6 ± 3.6	19.7 ± 2.2
Emetine	23.3 ± 3.9	29.1 ± 3.3
d-SOR		
Vehicle	38.8 ± 4.5	22.0 ± 2.5
Emetine	28.9 ± 4.0	19.1 ± 2.2
4*C*, left		
s-SOR		
Arc-MSO	20 ± 0.9	12.8 ± 0.7
Arc-ASO	16.5 ± 1.7	20.0 ± 1.8
4*C*, right		
d-SOR		
Arc-MSO	28.9 ± 3.0	17.3 ± 2.9
Arc-ASO	32.4 ± 4.7	18.1 ± 2.8
5*E*		
s-SLR		
Arc-MSO	36.5 ± 4.7	26.0 ± 3.6
Arc-ASO	39.6 ± 5.8	24.5 ± 2.0
l-SLR		
Arc-MSO	30.5 ± 3.8	19.6 ± 2.0
Arc-ASO	34.3 ± 4.3	24.6 ± 5.3
6*B*		
s-SOR		
5 min		
Arc-MSO	28.0 ± 3.2	18.0 ± 2.2
Arc-ASO	23.0 ± 2.4	21.9 ± 3.0
3 h		
Arc-MSO	25.66 ± 4.50	13.65 ± 1.26
Arc-ASO	28.96 ± 3.96	17.29 ± 1.92
d-SOR		
5 min		
Arc-MSO	41.8 ± 3.4	25.0 ± 2.4
Arc-ASO	38.1 ± 6.2	22.8 ± 3.2
8*B*		
xs-SOR		
Vehicle	32.3 ± 2.5	33.1 ± 4.4
BDNF	33.7 ± 4.8	22.0 ± 2.2
8*D*		
Vehicle		
Arc-MSO	25.8 ± 3.1	32.8 ± 3.4
Arc-ASO	28.2 ± 3.5	27.7 ± 3.0
BDNF		
Arc-MSO	32.3 ± 2.1	19.9 ± 3.0
Arc-ASO	29.1 ± 1.9	28.7 ± 2.0
8*F*		
s-SOR		
Same	37.4 ± 6.5	19.0 ± 4.0
Different	19.5 ± 1.6	24.0 ± 3.1

Results are expressed as mean ± SEM in seconds. Novel and familiar indicate to which of the two objects present during the choice phase the exploration time corresponds (novel location/identity object or familiar location/identity object).

**Table 4.** Total exploration times during the choice session of the SOR task

Figure	*p* value	*t*_total_
2*B*	0.1100	
Similar		40.66 ± 2.82
Dissimilar		47.77 ± 2.02
2*C*	0.3593	
Familiar		60.76 ± 4.30
Novel		53.1 ± 7.21
3*C*		T total
s-SOR	0.902	
MSO		50.66 ± 4.17
ASO		51.80 ± 8.92
d-SOR	0.354	
MSO		57.53 ± 5.41
ASO		62.47 ± 5.16
3*D*		
s-SOR	0.823	
MSO		51.23 ± 5.52
ASO		52.40 ± 6.26
d-SOR	0.077	
MSO		60.80 ± 6.51
ASO		48.01 ± 5.56
4*C*		
s-SOR	0.2059	
MSO		32.84 ± 1.16
ASO		36.47 ± 2.76
d-SOR	0.8750	
MSO		39.18 ± 3.21
ASO		38.65 ± 2.81
5*A*	0.174	
MSO		46.22 ± 5.05
ASO		50.50 ± 5.66
5*E*		
s-SOR	0.419	
MSO		63.61 ± 6.89
ASO		55.00 ± 6.38
d-SOR	0.310	
MSO		50.10 ± 5.28
ASO		58.95 ± 8.30
6*B*, upper		
s-SOR	0.837	
MSO		46.02 ± 5.13
ASO		44.92 ± 5.05
d-SOR	0.654	
MSO		65.28 ± 5.47
ASO		62.50 ± 7.70
6*B*, lower		
s-SLR	0.663	
MSO		39.31 ± 12.78
ASO		46.25 ± 5.39
8*B*	0.173	
MSO		68.37 ± 5.98
ASO		55.73 ± 6.41
8*F*	0.273	
MSO		56.48 ± 9.63
ASO		43.59 ± 3.50
8*D*	0.825	
Vehicle		
MSO		58.59 ± 6.47
ASO		55.88 ± 6.60
BDNF		
MSO		52.19 ± 3.93
ASO		57.85 ± 2.96

Results are expressed as mean ± SEM in seconds. *p* values are for the comparison between total exploration times during the choice session for each experimental group depicted in the same row. Paired *t* test was used for these comparisons, except in the case of Fig. 8*B*,*D*, for which unpaired *t* test and one-way ANOVA were used.

**Table 5.** Total exploration times during the sample session of the SOR task

Figure	*t*_total_
2*B*	
Similar	106.4 ± 6.16
Dissimilar	106.4 ± 6.73
2*C*	
Familiar	113 ± 14.20
Novel	97.81 ± 12.74
3*C*	
s-SOR	
BDNF-MSO	89.49 ± 8.86
BDNF-ASO	90.65 ± 14.98
d-SOR	
BDNF-MSO	73.97 ± 9.18
BDNF-ASO	78.33 ± 9.14
3*D*	
s-SOR	Veh
Vehicle	86.62 ± 8.58
Emetine	85.91 ± 12.36
d-SOR	
Vehicle	94.88 ± 9.26
Emetine	96.83 ± 11.71
4*B*	
s-SOR	
Arc-MSO	107 ± 9.56
Arc-ASO	114.7 ± 10.68
d-SOR	
Arc-MSO	108.7 ± 7.25
Arc-ASO	120.7 ± 5.62
8*A*	
Vehicle	120.7 ± 4.57
BDNF	114 ± 10.12
8*C*	
Vehicle	
MSO	108.3 ± 9.80
ASO	118.5 ± 10.97
BDNF	
MSO	129.5 ± 4.30
ASO	123.7 ± 8.86

Results are expressed as mean ± SEM in seconds.

To analyze whether Arc expression was required for this enhancing effect of BDNF, we combined injection of hrBDNF with Arc-ASO into Prh. Arc-ASO or Arc-MSO was injected 2 h before the sample phase, and hrBDNF or saline was injected 5 min after the sample phase (Fig. 8*C*). There were no differences in exploration time during the sample phase between Arc-ASO– and Arc-MSO–injected animals (Fig. 8*C*, bottom). Arc-ASO infusion, but not Arc-MSO infusion, prevented the BDNF-dependent enhancement in performance during the choice phase conducted the next day (Fig. 8*D*). In addition, one-sample *t* tests indicated that the only group with a discrimination ratio significantly above zero was the BDNF/MSO group (*p*_Veh/MSO_ = 0.0002, *t* = 9.47; *p*_BDNF/MSO_ = 0.03, *t* = 0051; *p*_Veh/ASO_ = 0.96, *t* = 3.01; *p*_BDNF/ASO_ = 0.9, *t* = 0.9). These results indicate that Arc expression is required for BDNF-induced increase in consolidation of highly overlapping memories.

### Molecular disconnection suggests that Arc is a critical effector of BDNF during discrimination of overlapping object memories in Prh

We next sought to determine whether BDNF and Arc interacted during consolidation of the similar SOR task. Thus, we conducted a molecular disconnection experiment. The rationale for this can be found in a typical brain disconnection experiment in which one wants to determine whether two brain structures are connected during a particular behavioral manipulation (Gaffan and Harrison, 1987; Ito et al. 2008). Assuming that the main connections between the two structures are ipsilateral, inactivation of the two regions in the same hemisphere would leave behavior intact, but contralateral inactivation would hamper performance. If, instead of two regions, we think of two molecular or gene expression pathways within a given structure, we can apply a similar line of reasoning. If the two molecular pathways interact to produce behavior, then blocking both of them in that region of one hemisphere would not have any effect, but blockade of one molecule in one hemisphere and the second molecule in the other hemisphere would produce a deficit.

Thus, to evaluate whether BDNF and Arc signaling pathways are connected in Prh, we blocked BDNF and Arc expression in the Prh of the same hemisphere or blocked BDNF expression in the Prh of one hemisphere and Arc expression in the Prh of the other hemisphere (Fig. 8*E*). We found no effect in the similar SOR task evaluated at 24 h if BDNF-ASO and Arc-ASO were injected into the same Prh, while injecting BDNF-MSO and Arc-MSO into the other Prh 2 h before the sample phase (Fig. 8*F*). However, when BDNF-ASO/Arc-MSO and BDNF-MSO/Arc-ASO were injected into Prh in different hemispheres, there was a significant impairment in the similar SOR task (Fig. 8*F*). There were no differences in total exploration times between the two groups (see Table 3). In addition, one-sample *t* tests revealed that the discrimination ratio from the “same” group was different from zero, whereas the discrimination ratio from the “different” group was not (*p*_same_ = 0.0023, *t* = 5.73; *p*_different_ = 0.29, *t* = 1.17). This result suggests that BDNF and Arc interact during consolidation of overlapping memories in Prh.

## Discussion

In this work, we have shown that BDNF and Arc are required for consolidation of overlapping object memories in Prh. Several of our results point at the BDNF–Arc pathway as an important player underlying disambiguation of overlapping object representations: (1) Both BDNF and Arc-ASO impaired memory only for the similar condition of the SOR task; (2) the effect of Arc-ASO is time restricted, suggesting that Arc is mainly involved in consolidation; (3) the amnesia caused by Arc-ASO is dependent on the delay between sample and choice, not affecting memory at short delays such as 3 h, but causing amnesia at 24 h; (4) Arc is expressed in an as-needed manner after encountering similar objects; (5) Arc in Prh is not required for acquisition/consolidation of overlapping spatial memories, indicating that these molecular processes in this structure are dependent on the type of representations that are necessary to solve the task; (6) the memory enhancement induced by hrBDNF is abolished completely by Arc-ASO, suggesting that Arc is one of the molecules required for the effect of BDNF; and finally, (7) BDNF and Arc molecular pathways interact during acquisition/consolidation of overlapping object memories as shown by the molecular disconnection experiment.

We used a modified version of the spontaneous object recognition task, and thus, there could be a concern regarding a change in motivation to explore the objects after a particular pharmacological manipulation (i.e., manipulations could change the animals’ preference for novel items to familiar items). In our experiments, this factor could not account for the differences in the discrimination ratios, because that would mean that our manipulations of the Prh somehow affected motivation only in the similar condition but not in the dissimilar condition. Moreover, the fact that infusion of the Arc-ASO 3 h after the sample phase did not affect novelty preference in the similar SOR condition effectively rules out the possibility that a change in motivation explains these results. Also, infusion of ODNs in Prh did not change exploration or cause memory impairment in a spatial object exploration task. In the experiment depicted in Fig. 3*C*, BDNF-ASO–treated animals showed a negative discrimination ratio. We have seen these type of results before using our spatial discrimination task (Bekinschtein et al. 2013), and it could be explained if the animals could not store separate representations of the two similar objects; then during the choice phase, it might seem that the novel object (made of two familiar features) would have been explored twice as long during sample, increasing familiarity during test.

These results indicate that BDNF and Arc take part in a protein synthesis–dependent mechanism important for consolidation of certain types of memories. This is remarkably similar to our findings in the DG of the hippocampus (Bekinschtein et al. 2013). Our results also suggest that there is interaction between BDNF and Arc during consolidation of overlapping object memories, indicating that Arc is likely an effector of the plasticity induced by BDNF. Importantly, we compared the similar and dissimilar conditions for all experiments, and the memory test was always conducted after the same delay for both of them (i.e., 24 h after acquisition). Because the effects were observed only for the similar condition, they were dependent on the similarity, but not on the delay of testing. Thus, these mechanisms are specifically involved in discrimination of overlapping memories, but not on their persistence. However, we cannot conclude from these results that BDNF and Arc are not involved in the mechanisms of longer-lasting maintenance of nonoverlapping memories in Prh or that other known plasticity molecules such as Zif268 are required for consolidation of nonoverlapping memories in this structure.

There is convincing evidence to indicate that Prh, rather than storing simple features of objects, stores conjunctive representations that can later be used to disambiguate particular objects during memory retrieval. This hypothesis has been previously tested by examining the role of Prh during discrimination of objects that shared overlapping features at the moment of retrieval (Norman and Eacott, 2004; Bartko et al. 2007). In this sense, Prh could be thought of as a structure that acts as a “pattern separator” for representations of objects, disambiguating overlapping information into separate and less confusable representations. In fact, recordings of single units from the Prh showed populations of neurons whose firing rate changed gradually as the originally learned objects were ambiguously morphed to varying degrees, and other neurons whose firing rate changed abruptly according to the rewarded response categories associated with the objects. They suggest that this abrupt change in the firing rate could be a result of the orthogonalization of the original morphing continuum (Ahn and Lee, 2017). This neural perirhinal population with orthogonalized responses that correlate with their memory-guided choices could be the neural substrate that supports the consolidation of similar objects into nonoverlapping representations that guide behavior in the SOR task.

Our experiments suggest that, at least for storage of object representations, but not of spatial representations, BDNF and Arc are essential for consolidation of separate memories and a part of a time-restricted, protein synthesis–dependent mechanism of memory stabilization in Prh. These results are in line with the evidence indicating that structures in the medial temporal lobe are specialized in processing different types of representations. Because the Prh receives prominent afferents from the ventral visual stream (the “what” pathway), it has been suggested to be at the top of a hierarchical network of object processing (Kent et al. 2016). This idea is compatible with the thought of Prh being a pattern separation structure. On the other hand, the postrhinal cortex (Pc) lies posterior to the Prh and receives afferent projections primarily from the dorsal (“where”) processing system (Suzuki and Amaral, 1994) that has been implicated in visuospatial processing (Kravitz et al. 2011). Because the “what” and “where” features are essential to episodic memory, information from Prh and Pc has to be integrated into an experience. In fact, efferents from these structures project preferentially to different regions of the entorhinal cortex (EC), which, in turn, project to the hippocampus (Witter, 2007). Although Prh primarily projects to the lateral entorhinal cortex (LEC), the Pc projects to the medial entorhinal cortex (MEC; Suzuki and Amaral, 1994). This pattern of connectivity suggests a segregation of object and spatial information processing in EC that could be integrated within the EC or in the hippocampus via the perforant path (Witter, 2007). Thus, plasticity in the Prh could occur at the synapses connecting to the LEC, facilitating object information processing necessary for episodic memory. It is highly unlikely that our manipulation of Prh, such as infusion of ASO, reached Pc, since the infusion site was far away from this structure, and we observed no spreading of the oligonucleotides outside Prh.

It is widely believed that changes in synaptic strength support long-term memory storage in the brain (Kandel, 2001). *In vitro* studies have found that Prh neurons can develop both long-term potentiation (LTP) and long-term depression (LTD; Bilkey, 1996; Ziakopoulos et al. 1999; Cho et al. 2000; Massey et al. 2001). *In vivo* experiments have strongly associated object recognition memory with LTD induction and maintenance in Prh (Griffiths et al. 2008). This type of plasticity has been found to be dependent on internalization of AMPA receptors in Prh. In this sense, Arc KO mice have deficits in many learning tasks, including object recognition, and they have diminished LTD in the hippocampus (Plath et al. 2006). In another study, Jakkamsetti et al. (2013) observed that Arc-expressing neurons preferentially develop LTD in response to activation of group I metabotropic receptors in CA1, and that this molecule is required for mGlurR-dependent LTD. It is possible that similar mechanisms are involved in Arc-dependent consolidation of overlapping object memories in our behavioral paradigm. Arc has been implicated in AMPA receptor trafficking at the synapses (Rial Verde et al. 2006; Shepherd et al. 2006; Waung et al. 2008); thus it seems logical that this could be a possible mechanism for object memory storage in Prh.

One previous study used BDNF-ASO to block BDNF expression in Prh either before or after the sample phase in a spontaneous object recognition paradigm (Seoane et al. 2012). BDNF-ASO injected 1 h before or immediately after acquisition impaired familiarity discrimination at 24 h, but not 20 min, after acquisition. Infusion of the ASO 6 h postacquisition did not impair memory 24 h later. However, we believe the results of our study do not generalize to the molecular mechanisms of recognition memory but rather the mechanisms underlying storage of unique representations of objects in Prh. In our experiments, we found a memory impairment caused by BDNF-ASO only in the similar, but not in the dissimilar, condition. Our results are consistent with a role of Prh in storage of nonconfusable object representations.

Given that adult neurogenesis in the DG has been implicated in the discrimination of overlapping spatial representations (Clelland et al. 2009; Kheirbek et al. 2012; Nakashiba et al. 2012; Bekinschtein et al. 2014b) and that adult neurogenesis is absent in Prh, it is clear that the underlying cellular mechanisms of pattern separation are different between structures such as the DG and Prh. However, despite these anatomic differences, several molecular mechanisms that influence plasticity changes at synapses seem to be similar and common to memory storage processes. Synaptic mechanisms for memory consolidation are widely conserved across species despite the differences in their brain anatomy. Molecules such as cAMP response element–binding protein (CREB) are essential in consolidation of many types of learning in invertebrates and vertebrates (Carew and Sahley, 1986; Abel and Lattal, 2001; Schafe et al. 2001; Barco et al. 2006), and compounds such as BDNF are important parts of the machinery involved in plasticity of many sorts, from synaptic plasticity and memory to development and pain (Lu and Chow, 1999; McAllister et al. 1999; Bramham and Messaoudi, 2005; Pezet and McMahon, 2006; Bekinschtein et al. 2008). Thus, from an evolutionary perspective, it seems logical that different regions of the brain became specialized to process particular types of representations, but the underlying plasticity mechanisms were conserved. In light of this argument, it makes sense that some of the main players in the intracellular molecular plasticity mechanisms driving consolidation of overlapping memories appear to be identical across different brain regions. Adult neurogenesis, therefore, might have evolved at least in part as a cellular mechanism that prevents interference specifically between spatial and episodic representations—and not representations involving only objects—because the increased excitability and plasticity of adult-born neurons in the DG is necessary for the processing of highly complex information present in places and episodes.

To our knowledge, the present study is the first to provide evidence regarding the molecular pathways involved in the consolidation of overlapping memories outside the DG and, together with our previous studies, to demonstrate that BDNF is an important plasticity molecule involved in this process in multiple brain regions. In addition, we show, for the first time, that under certain conditions Arc protein is required for spontaneous object recognition in Prh and, in particular, for storage of separated representations of overlapping objects. Our results point toward an evolutionary convergence of the molecular mechanisms involved in plasticity required for storage of unique representations across different regions of the brain. Importantly, these molecular mechanisms are not general to all conditions of object (or location) recognition; they were required only when similar memories had to be kept distinct.

## Synthesis

Reviewing Editor: Morgan Barense, University of Toronto Decisions are customarily a result of the Reviewing Editor and the peer reviewers coming together and discussing their recommendations until a consensus is reached. When revisions are invited, a fact-based synthesis statement explaining their decision and outlining what is needed to prepare a revision will be listed below.

Although I found the response to the concerns raised by the reviewers thorough and convincing, I was concerned that there were several instances in which a concern was addressed only in the letter, with the clarification never making its way into the manuscript. This was likely exacerbated by the fact that the line references in the response to reviewers are nearly always inaccurate, but I still believe that there are several important points raised (and addressed) in the letter, but not in the manuscript.

By way of example, here is a non-comprehensive list:

- The discussion on page 2 regarding BDNF-ASO reducing steady-state levels at the time of the sample phase.

- Page 4-5: Addressing the reviewer's confusion regarding ODN concentration

- Page 5: The discussion of hr-BDNF administration and Arc expression enhancement

- Page 10: Clarifying the misunderstanding regarding exploration time differences across groups.

I ask that you submit a revised manuscript and response letter that clearly indicates exactly where each point was addressed in the manuscript. If the authors do not wish to change the manuscript in response to a reviewer's concern, please let me know your reason for doing so.

I also note that the reviewers commented on some organizational issues regarding the figures and tables. While it seems that the figures have improved considerably, I still found the tables difficult to follow at first glance. The caption should describe all components of the tables. For example, it's not immediately intuitive what the left-most “Fig n” column depicts. It should be directly stated what statistical comparison was used to generate the p-values depicted in Table III. For Figure 7C, I suggest writing out the full words for “C”, “S”, and “D”, as there appears to be sufficient space. If not, please clearly define these abbreviations.

## Author Response

Response to reviewers

We thank the reviewers and the Editor for their input which, we believe, has substantially improved our manuscript. Here you will find a point-by-point response to all the reviewers' concerns. Changes to the manuscript are highlighted in red.

Reviewer #1 thinks that “The most interesting finding of the manuscript is the convincing evidence that Arc in the perirhinal cortex is functionally necessary for the consolidation of pattern-separated object memories. This is a novel demonstration of plasticity mechanisms in the PRh.”

We are pleased the reviewer finds our Arc evidence convincing and important. Indeed we additionally show that Arc is part of the machinery of pattern separation and a likely a donwstream target of BDNF, which has not been previously shown in Prh. However, we think the overarching finding of the similarity of mechanisms of pattern separation across very different brain regions in very different menmonic domains is the finding that will most greatly change people's thinking about pattern separation, and that is why we have emphasised that aspect of the study in our article (for example, see lines 77 to 98 on the revised version). While our study provides some confirmatory (see our several previous studies), as well as some novel information on novel object recognition mechanisms of consolidation, the main aim of our study, and the evidence and novel information provided by it, are vastly more interesting than that. Specifically, we are studying the mechanism of pattern separation - the ability to keep memories from being confused with one another - which has become of great interest to researchers and which is assumed by the majority to be the sole province of the hippocampus. He we confirm a role for Prh in pattern separation, but most importantly, show that mechanisms of pattern separation in Prh are the same as those in the hippocampus. In light of the predominant ‘hippocampocentric’ view of pattern separation, this finding will be surprising to many, and will change the way researchers think about pattern separation. Importantly, we provide controls to show that these mechanisms are required only when pattern separation is required, by holding the delay (the traditional manipulation of memory load or memory persistence) constant

The experiments are well-designed, appropriately analysed and, on the most part, accurately interpreted. I remain a bit confused about the overall structure of the manuscript (although it is certainly the authors' decision to make). To me, the strongest evidence within the study relates to the Arc experiments, yet everything is contextualised within a BDNF story.

We hope it is now clear from the above that the study is best understood not as a ‘BDNF storyߣ. BDNF is discussed because this a mechanism of pattern separation that we found necessary in both the hippocampus (Bekinschtein et al., 2013), and the perirhinal cortex (see lines 77 to 98, 655 to 663 and 729 to 745).

The BDNF and emetine data do not show direct evidence for a role in consolidation, as treatments are given pre-learning and there are no tests of short-term memory. There is relatively little that can actually be concluded about the role of BDNF. There remains an assumption (perhaps justified) that the action of BDNF ASO to impair the acquisition/consolidation of overlapping memories is due to its effect to knock down BDNF expression. The kainic acid experiment does not show that BDNF knockdown is the only effect of BDNF ASO, and there is no evidence presented to show that the acquisition of overlapping memories engages BDNF expression. Nor does it show that BDNF ASO attenuates such upregulation. This leads to a different question, which is whether it is upregulation of BDNF that is necessary, or pre-existing expressed BDNF. Presumably, the timing of ASO might impact upon both, and an expression experiment would have been useful to disambiguate. The picture for Arc is a lot more convincing. At the very least, the interpretations and discussion of the BDNF and emetine data need to be more nuanced. For example, given that ongoing protein synthesis has recently been shown to be necessary for memory retrieval, there remains the possibility that the same is the case for acquisition. Without a short-term memory test, or post-acquisition infusions, the conclusion should be toned down.

Here the reviewer makes a good point regarding the role of BDNF in consolidation, and that testing short-term memory after pre-training BDNF ASO infusion would be informative in this case, as it has not been evaluated in this version of the behavioral paradigm. To do this, we injected BDNF ASO or MSO into Prh and evaluated short-term memory in the similar version of the task. We did not find a significant difference between ASO and MSO. Both ODNs were infused 2 hr before the sample phase and memory evaluated 3 hr post-acquisition. We found that both groups remembered equally (BDNF MSO DI 0.23{plus minus}0.03 vs. BDNF ASO DI 0.24{plus minus}0.03, n=7). Since short-term memory is not affected, we believe it makes a stronger case for BDNF involvement in consolidation. We added this information in lines 411-417.

It is unlikely, however, that BDNF-ASO reduces steady-state levels at the time of the sample phase. Previous experiments using fear learning have shown an amnesic effect on long-term memory of pre-sample BDNF blocking antibodies, but not of BDNF-ASO, suggesting that BDNF-ASO only acts on de novo BDNF synthesis (Slipczuk et al, Plos One). Although in this work we did not perform a dose-response curve of BDNF-ASO on BDNF protein levels, previous work showed that 2h post injection, there were no differences in BDNF steady-state levels between BDNF-ASO and BDNF-MSO in the dorsal hippocampus (Bekinschtein et al, 2007, Neuron). This also suggests that in these experiments, BDNF-ASO blocks BDNF expression induced by learning. We have now clarified this in the manuscript (see lines 393 to 401).

There is repeated reference, in the abstract and introduction to statements such as “Although molecular mechanisms have been shown previously in PRh, these have always been dependent on delay, suggesting a role specifically in persistence.” First, these statements are never supported by any citation. Second, it is not clear to this reviewer what is actually meant. Third, I'm not actually sure that there is any evidence in the presented data to support the statement in the abstract that “BDNF and Arc involvement were independent of delay”. Again, I'm not clear what this actually means. Moreover, in the Arc experiments there is a delay-dependent effect, which is correctly interpreted as likely reflecting an impairment of consolidation. I suspect that this repeated emphasis is related to the historical literature on rhinal cortex involvement in memory, but it could be explained much more clearly, and I personally find it distracting to the main finding of the study.

Again, we are sorry we were not clear enough on this issue, and we now explained our aims better in the revised version. We compared the similar and dissimilar conditions for all experiments and the memory test was always carried out after the same delay for both of them (i.e. 24 hr after acquisition). Since the effects were observed only for the similar condition, they were dependent on the similarity, but not on the delay of testing. It's true that Arc ASO only affects memory evaluated at 24 h, but the independence of the delay refers only to the comparison between similar and dissimilar conditions, a specific effect on pattern separation, which is what the work is about. We will try to make it clearer throughout the MS. The main finding of this study is that BDNF and Arc are required for consolidation in Prh only for similar object memories, not their involvement in consolidation of object memories independently of their similarity. We clarified this in the discussion (see lines 655 to 663). We added some references to the statement that the molecular mechanisms described previously were dependent on the delay of testing (see lines 89 to 91).

The discussion makes the argument that “different regions of the brain became specialized to process particular types of representations, but the underlying plasticity mechanisms were conserved. In light of this argument, it makes sense that some of the main players in the intracellular molecular plasticity mechanisms driving consolidation of overlapping memories appear to be identical across different brain regions.” How can these be reconciled with the observations that BDNF and Arc are functionally involved in other forms of plasticity and memory that do not involve pattern separation. Is there reason not to go further and conclude that the main players in the intracellular molecular plasticity mechanisms driving consolidation of any memory appear to be conserved across different brain regions. I don't see any evidence for plasticity in a given brain reason being shown not to involve Arc, or that it has a special involvement in pattern separation.

Once more, we apologize we were not clear enough. We have somehow given the impression that we think BDNF or Arc are important only for pattern separation. Indeed we agree with the reviewers' take that the main players in several aspects of memory including pattern separation are conserved across regions. The revision makes this all much clearer. We cannot assume that the only memory processing that Prh does is pattern separation. In our opinion, this conclusion would exceed the data presented here. Plasticity in different structures can rely on distinct molecular mechanisms, for example LTD in Prh required both NMDA and mGlu receptors, while in the DG requires either NMDA or mGlu. We don't believe that should be a given fact that all consolidation mechanisms require the same molecules. For example, ZIF268, another immediate-early gene, seems to be involved in consolidation of fear conditioning in the amygdala (Malkani et al, L&M, 2004), but not in contextual fear conditioning in the hippocampus (Lee et al, Science, 2004). We believe such conclusion would mean an overgeneralization of the phenomenon observed here. We added a paragraph in the discussion to clarify this issue (see lines 660 to 663).

Other comments:

- The abstract would benefit from reference to the use of antisense, to save the passing reader having to delve into the methods.

Thanks for pointing this out, a reference was added.

- Some greater accuracy in statement would also help. E.g. in the abstract it is stated that “This is the first time that Arc has been implicated in...”. It is certainly not the first time that Arc has been implicated in object recognition memory, as already cited in the manuscript. It may well be the first time Arc has been implicated in pattern separation.

Thank you, this was corrected.

- The manuscript needs to be thoroughly proof-read so that it conforms to the Journal of Neuroscience structure of the methods being presented before the results. Please pay particular attention to definitions of acronyms and explanation of the tasks. Currently, the methods only really make sense once the results are read. Moreover, there seems to be an excessive amount of methodological detail in the results section. On a related note, the methodology could refer helpfully to the relevant figure panels that represent the experimental timeline and approach.

We apologize for this. The manuscript has been thoroughly proofread to conform to the structure for the journal. Methodological detail will be transferred from the results to the ‘methods’ section and appropriate figure numbers will be indicated.

- The experimental design and statistical analysis section could helpfully include the statistical methodology for the sample phase and also the use of one-sample t-tests.

Thank you for pointing this out. Statistical methodology for the sample phase was included in the ‘statistical methodology’ section. We run one-sample t tests for the data from the test phases. All MSO-injected animals showed significant p values compared to zero. The rest of the discrimination ratios of ASO-injected animals were not different from zero, except for the experiments depicted in figure 3C, which showed a negative discrimination ratio. We have seen these type of results before using our spatial pattern separation task and it could be explained if the animals could not store separate representations of the two similar objects, then during the choice phase, it might seem that the novel object (made of two familiar features) would have been explored twice as long during sample, increasing familiarity during test (see lines 644-649).

- It is not made clear why a lower concentration of ODN was used for the analysis of spread after injection. I could speculate, but it would be helpful for the rationale to be made explicit.

We understand this was not clear enough and we can easily explain it. We always used the same concentration of ODN, we injected 2 nmol/side. Maybe the confusion arises because the concentration in the ‘Methods’ section is expressed as 4 nmol μl-1/0.5 μl side. The concentration of the solution was 4 nmol/μl, but the injection volume was always 0.5 μl. We have clarified this in the “methods” section (line 150).

- Please include explicit references to justify the statement that other tests of pattern separation.

Thanks, references were included

- I am somewhat surprised that there is no attempt to show that hr-BDNF administration enhances Arc expression.

The reviewer made an interesting observation. It has already been shown that hrBDNF induces Arc expression (Ying et al, JoN,2002, Lee et al, Science, 2004). We felt that the functional experiment in which Arc-ASO blocked BDNF-dependent enhancement was much more convincing than a correlation. We have added this information in lines 559 to 561.

- There are some inconsistencies in the statistical presentation in the figure legends that are distracting to the reader and some full statistics are missing (e.g. Fig 2C).

We are sorry for these mistakes. Figure legends were corrected so that statistical analysis are consistent with the data presented.

- Data are actually presented as means + SEM

- Why are the SLR data presented as media + SEM, while the SOR data are presented as means + SEM?

Thank you for detecting this mistake. Data was always presented as mean+SEM. This was corrected.

Reviewer #2 thinks “This manuscript provides new results that will be of interest to persons working on the circuitry and mechanisms of encoding, and the topic of pattern separation in the perirhinal cortex is both interesting and not well investigated.”

However there are many distracting organizational issues which impaired the presentation of the results (as noted in detail below) and other issues that weakened some of the major conclusions (e.g., the lack of negative control regions for treatments with the ASO.

Also “This manuscript provides new results that will be of interest to persons working on the circuitry and mechanisms of encoding, and the topic of pattern separation in the Prh is both interesting and not well investigated.”

Manuscript organization. The paper was rather hard to read for a number of reasons. First of all, SOR and s-SOR, d-SOR were not defined at first appearance. These abbreviation should have been defined at first appearance in the Methods section: SOR and SLR abbreviations are first used in the ‘Apparatus’ section of the Materials and Methods however they are not defined until the ‘Experimental Design’ section.

We apologize for this. It was corrected.

Second, the figures are referred to out of their order of appearance and, even for a given figure, the panels are referred to out of order - and there was no apparent reason for this - it could have been avoided.

Thank you for pointing this out. We modified the text and figures so that information appears in the order of appearance in the MS.

The tasks should have been illustrated in an early figure in association with presentation of the Methods section text.

This is a good point and will make the MS easier to read. An illustration of the task was added to the Methods section (see Fig. 1).

In addition, the objects should be shown: we are relying on the authors comment that items are similar or not similar but do not see those objects. This is a very important element in the manuscript and the materials should be shown.

We agree this would be important for the readers. Examples of objects are now shown in the Methods section (see Fig. 1).

Third, the illustrations would benefit from headings on the graphs (or perhaps more information on the Y-axis such as ‘s-SOR discrimination index’) to make it easier to appreciate the results. Other organizational elements (details) are noted in “Minor points” below.

Thank you for pointing this out, more information was added to the Y-axis. Due to space limitations headings were not present in all graphs. However, in the figures in which similar and dissimilar conditions were compared, the similar condition is always on the left panel and the dissimilar on the right. Headings are already present in the scheme of the tasks for each figure.

A few major issues arose with either the experimental design or the absence of justification for the design chosen

1. Why weren't BDNF and ARC levels measured after the similar and dissimilar spontaneous objection recognition tasks? These are the circumstances being compared.

The reviewer makes an interesting observation. We did measure Arc levels after similar and dissimilar conditions. BDNF levels were proven difficult to measure, because of its low expression in Prh. Nonetheless, BDNF-ASO only caused amnesia for the similar condition, indicating that synthesis of BDNF is only required to consolidate pattern-separated memories. It is unlikely that the ASO diminishes BDNF steady-state levels (see response to Reviewer #1). we added this information in lines 559 to 561.

2. Why was such a different task used (i.e. an object is removed and location is changed for one of the objects) for the experiments in Figure 7 rather than just shortening the acquisition time to make the original task used throughout the paper more difficult? It is hard to draw the same conclusions from with the altered task.

We apologize we were not clear enough throughout the MS regarding the critical question (see response to Reviewer #1and lines 77 to 98, 655 to 663 and 729 to 745 ). We modified the MS for more clarity. We are happy to explain the reasons why we chose this particular task. The critical manipulation in all of these experiments was the similarity of the objects and not the length of time the animals had to acquire object information. We were addressing whether hrBDNF could enhance pattern separation, not whether it could make a weak memory more persistent. Persistence is not the process we are studying in this work. There is the possibility that the reviewer assumed that the critical variable was the difficulty of the task, but this was not the question we were asking. In fact, increasing the load on pattern separation in a spatial task, did not engage Arc in Prh, showing that it was not the difficulty of the task what made it dependent on BDNF and Arc, but rather the similarity and domain of the to-be-remembered items.

3. There should be more discussion of the circuitry thought to be involved in encoding object vs spatial information. There is a real literature in this area with perirhinal cortex being specifically implicated in object recognition as opposed to encoding spatial information. Along these lines it is unclear why the spatial task was chosen for the experiment illustrated in Figure 4D-E. Would a lesion or inactivation of the Prh disrupt this task (independent of the specific chemical targets)? Has this been done?

The reviewer is correct, there is literature in this area regarding the involvement of Prh in object processing and not in spatial processing. However, most of the studies involve lesions and none of them involve manipulating the expression of plasticity molecules. We have now discussed carefully the role of Prh in object, but not spatial memories (see lines 683 to 702), but, again, this was not the aim of this particular study, since, as the reviewer mentions, it has been determined in many other studies. As far as we know, there is no information regarding the effect of lesions or inactivation of the Prh in this exact task.

4. An argument for the involvement of BDNF/Arc specifically within Prh would have been stronger if the same manipulations were applied to postrhinal cortex (or another putative ‘control region’ for object recognition) or some area involved in spatial tasks. This one is important.

A control region would be important if the question were whether Prh was specifically involved in discrimination of objects. But this has already been shown and is part of the literature the reviewer alludes to above. For example perirhinal cortex has little involvement in spatial tasks, no matter how difficult, and hippocampal lesions have been tested explicitly on object pattern-separation tasks and were found to have no effect (e.g., Winters et al, 2004; Saksida et al., 2006). Indeed, the major conclusion of our study is that the same molecular mechanisms operative in the hippocampus for pattern separation of locations are operative in Prh for the pattern separation of objects. The extension of this conclusion is that other regions - for example postrhinal cortex - will also use these same mechanisms for pattern separation of whatever stimuli that region represents, and this would be very interesting to explore, but would require its own set of experiments. Indeed one can think of our previous hippocampal BDNF study (Bekinschtein et al, 2013) as a control region for the present study and viceversa - but the variable that distinguished the structures' involvements in pattern separation was not the molecular mechanisms, but the stimuli the structure represents. In addition, the postrhinal area is not close to the infusion site we have used in this study. Given the spread of the ASO, it is highly unlikely that it reached the postrhinal cortex.

5. Comparisons are continually made to the dentate gyrus LTP and signaling mechanisms, this discussion needs to be expanded to include evidence that the lateral and medial perforant pathways (responsible for object vs spatial information) have different mechanisms for LTP.

Thank you for this suggestion. We added a paragraph discussing the role of the lateral and medial perforant pathways in object vs spatial information (see lines 683 to 702).

Minor Points that can be addressed by changes to presentation.

1. The ‘Subjects’ section of the Materials and Methods states that the animals are food deprived to 85-90% of their free feeding weight, however no reason is given for the food deprivation as the tasks do not involve food reward.

Thanks for pointing this out. We clarified this issue in the manuscript. We have observed that food deprivation increases spontaneous exploration of the objects making the task more robust (see line 124).

2. At the end of the ‘Immunoblot assays’ portion of the Materials and Methods section it states that data depicted in Fig 6D was transformed, however there is no D in Fig 6.

We apologize for this mistake. This was corrected, the data referring to is depicted in Fig 7C.

3. The Methods need to be expanded for clarity. specifically:

-In all tasks that have a 2 variations the objects are depicted as letters and the same letters are used, does this mean that the same objects are used even though the same animals are run on both tasks? If this were true it would alter the interpretation of the data. (beyond this the letters on some of the objects cannot be read - they are too small).

This can be easily clarified. We always used different objects for our within subject design. We clarified this in the ‘Methods’ section (see lines 203, 204 and 240 to 243). Due to space limitations it would be unpractical to increase letter sizes within the scheme of the task. The letters corresponding to each different condition will be clearly stated in Methods and again in Results, and also each figure already has additional headings on the graphs to indicate the version of the task that was used.

4. The tables are very confusing and need to be modified to be more consistent for clarity. Additionally, statements like on line 352 refer to significance (or lack of significance) in the tables however p values are only given in Table 3. All tables should be updated to include p values or at least to indicate whether comparisons that are not shown in the figures are significant - with some mark denoting significance level (p>0.01, p>0.001, etc).

Thank you. We can clarify this issue. Significance is reported for the discrimination ratio, which is a much more reliable variable than absolute exploration times for each of the objects, reported in Table II. The discrimination ratio as we have calculated is standard in behavioural studies, and used in a number of different behavioural paradigms. We decided to include additional information in Table II to illustrate the actual time the animals spent exploring the objects, which cannot be inferred from the discrimination ratios. All relevant p values are reported in the figure legends. Table III compares total exploration times during choice for ASO- and MSO-injected animals. These values are compared to show that the treatment did not significantly affect general exploratory behavior, but just how they divided exploration time between novel and familiar objects (indicated by the discrimination ratio). We have added information in the table legends to clarify the data and statistics.

-Additionally in many cases the data do not match the figures. For example in Table 2 it refers to Figure 2D-E and 2F-G, however Figure 2 only goes up to letter D.

We apologize for this mistake, it was corrected.

5. The control task included in Figure 1D needs to be clarified, there is no clear explanation for this task in the methods, and when it is referred to in the Results section on lines 363-367 the object comparisons are different than what the figure is depicting.

We were probably not clear enough regarding this control task. We explained better in the MS why we used it. Briefly, since this is a novelty exploration task, we needed to verify that the novelty coming from a change in the number of objects between the sample and choice phases was not driving exploration, but rather the novelty of the object identity. We clarified this in lines 363-364. We corrected the MS so that the letters indicating the objects are consistent between the text and the figure. Thank you for pointing this out.

6. Line 437 states that there was no difference in exploration time between the two groups and refers to Table 3, stating that acquisition of the task is not effected. However this should be table 4 and p values are needed to support the conclusion

This is a misunderstanding, we can explain. There were no differences in exploration times during the choice phase between ASO- and MSO-injected animals. In addition, the p values are present in the text in line 450. Table III indicates total exploration times during choice and p values are indicated. Table IV reports exploration times during the sample phase. We have modified the table legends to clarify these issues.

7. For clarity the panels in figure 4 (e.g. A-D) should be arranged to follow the order of presentation of the different data points in the results section.

Thank you for pointing this out. We arranged the panels so that they follow the order of presentation.

8. Line 669: ‘we’ should be ‘were’

Thank you, this was corrected.

9. There are many inconsistencies with the statistical representation (both written and graphically) for the same type of data (i.e. discrimination index) in all cases it should be assessed whether the DI is statistically significant within each group to indicate learning, and then group differences should be considered. In many cases only group differences are assessed.

We run one-sample t test to analyze learning for each group and we included them in the revised version of the MS. In all cases of MSO-injected animals, p values were significant. Significant values were also obtained for the discrimination ratios of ASO-injected animals that did not differ from MSO-injected animals. The rest of the discrimination ratios of ASO-injected animals were not different from zero, except for the experiments depicted in figure 3C, which showed a negative discrimination ratio. We have seen these types of results before using our spatial pattern separation task. It could be explained if the animals could not store separate representations of the two similar objects, then during the choice phase, it might seem that the novel object (made of two familiar features) would have been explored twice as long during sample. We have added this to the manuscript (see lines 644-649 and response to Reviewer #1)

10. Abbreviations used in the figures should be defined and used consistently in the figure legends.

We are sorry for this mistake, we checked the MS for consistencies in the use of abbreviations.

11. There are no asterisks on Figure 2C showing significance, which does not match the figure legend.

We apologize. we added the asterisks, they were lost during the editing of the figure.

12. It seems like Figure 4 A-C would fit better in Figure 3 to make it more consistent with Fig 2.

We understand that this would make sense. However, merging these two figures would make it more difficult to read. As Reviewer #1 said, letters are already small, if we did this, letters would be unreadeable.

13. In the figure legend for Figure 5B it states the p value as 0.046 however ** are used to show significance on the figure. This level of significance would typically be denoted by one star.

-Also in Figure 5's legend ‘Data are expressed as mean +/- SEM is said twice’.

Thank you for pointing this out. We corrected these mistakes.

14. In the legend for figure 6 and 7 p values should be given for the post hoc tests.

Asterisks within the figures indicate p values after post hoc tests. Level of significance is stated in the figure legends.

15. Line 442-443 states that, ‘no change in performance was seen after administering Arc-MSO’ however this should be reworded because that was not directly tested.

This is a good point. This sentence was changed to “These results cannot be explained by nonspecific damage to Prh by the oligonucleotide Arc-ASO, because no change in performance was seen after administering Arc-MSO, which has the same base composition, and staining did not reveal any lesion to the site of infusion” (lines 471-473).

16. in the abstract on line 66 it would be helpful if they specified that “.. Prh BDNF...” and on line 67 specified I“..is required for separable storage...”

Thank you, this was corrected.

17. the introduction gives the results in too much detail of the results - the last paragraph should be shortened and allude to results without giving in detail

The last paragraph was shortened to allude to the results without giving too much detail.

18. The sentence on line 117 should be rewritten: the ‘for example” does not lead to things that illustrate the point of the first part of the sentence

Thank you for pointing this out. The sentence was rewritten to include “for objects in Prh”.

19. wording on the sentence beginning on line 350 is repeating information given earlier in the manuscript - not needed

This sentence was deleted.

20. To consider the arguments on timing it would be helpful to know how long the ASO acts - in suppressing things like Arc expression

We can give some information. Infusion of Arc ASO can decrease steady-state levels hours after the injection (Guzowski et al, JoN 2000). The timing of infusion was conducted as previously described for this and other ODNs. The pre-sample time was chosen because ODNs are slowly taken by cells, so for them to have an effect on de novo synthesis they need to be injected at least 1,5 h before the experience. Thus, the ODNs inhected 3 h post-sample might affect protein synthesis at around 4,5 h post-sample, when consolidation seems to have ended. These results are similar to the ones obtained when infusiing Arc-ASO into the amygdala to block fear extinction (Onoue et al, 2014), pre-extinction infusion caused an inpariment, but infusion 3 h post- extinction training did not produce any effect. We added this information in lines 517 to 525.

21. some statements in the results provide too much interpretation for the ‘results’ section in my opinion: these major conclusions should: be in the discussion: e.g., lines 406-407. Also text on lines 440-441 (Plus the argument here seem circular...if consolidation phase is defined by the period during which the memory trace is disruptable than any effect of treatment could be concluded to be disrupting ‘consolidation). To get away from the issue of a potentially circular argument they need to define the consolidation phase by some other criterion than the present results.

This is a good point. We moved some of the statements from the results to the discussion section (see lines 634 to 644). Regarding the circularity of the argument, it is not true that any effect of treatment could be concluded to disrupt consolidation. Pre-sample treatments could be affecting initial learning, and that is exactly what we tested with the experiment in which we evaluated short-term memory.

Reviewer #3 believes “The paper by Bekinschtein et aldocuments a comprehensive set of experiments showing that blocking the expression of BDNF, Arc or their interactions impairs rats’ abilities to discriminate between novel and familiar objects that share features. This is potentially interesting work and the authors should be commended for the large set of experiments that were included to examine potentially critical molecular mechanisms within the perirhinal cortex for discriminating between similar objects.”

Main concern:

First, it is problematic that the authors use the term pattern separation in the context of their results and conclude that Arc and BDNF are critical for perirhinal cortical pattern separation. As the authors correctly define, pattern separation is a computational process by which small differences in inputs are amplified into larger differences in outputs (Marr, 1971). It has effectively been argued that the original definition of pattern separation as a computational process must be parsed from its newer, colloquial definition as a form of behavioral discrimination. More specifically, as written by Santoro (2013, Frontiers in Behavioral Neuroscience):

... behavioral pattern separation is consistent with pattern separation of cell ensembles, but it does not entail it. This is an important distinction, as it implies that behavioral pattern separation tasks cannot be used as correlates of, or cannot be used to infer the existence of cell ensemble pattern separation until direct evidence for the causal relationship between cell ensemble pattern separation and behavioral pattern separation is established. Thus, to demonstrate that the perirhinal cortex does perform this computational process, and that it relies on Arc and BDNF, one must be able to show the input to perirhinal cortex from sensory areas has high overlap that is reduced in the projections to entorhinal cortex and hippocampus and then test the molecular mechanisms of this computation. Inferring that the perirhinal cortex is performing pattern separation based solely on behavior and overlapping molecular mechanisms with the dentate gyrus is completely insufficient. Notably, the Stark lab changed the name of a task they use for assessing similar behavior from the “Behavioral Pattern Separation Task” to the “Mnemonic Similarity Task.” Thus, there is an emerging understanding in the learning and memory field that behavior alone cannot be used as a proxy to infer neural computations. That being said, these data are still useful and interesting, but the authors should interpret them in the context of behavioral discrimination and not pattern separation

We agree this is an important point. It is true that we did not do any experiment in this study that shows that the inputs are less correlated than the outputs at the neuronal level. However, we believe the term “discrimination” does not fully describe the results we have obtained. Discrimination occurs for both similar and dissimilar objects, but our manipulations of the Prh only have an effect on separation of similar objects. Thus, we prefer to use “discrimination of overlapping memories” or “discrimination of overlapping representations” when referring to the ability to behaviourally separate similar representations into separate memories. Thus, we have changed the title of our MS and replaced the term “pattern separation” in the text accordingly.

Along these lines, a recent paper has shown that the firing properties of perirhinal cortical neurons change nonlinearly as one stimulus is morphed into another (Ahn and Lee, 2017, Cerebral Cortex). These data are extremely relevant to the current paper and should be discussed.

Thank you for pointing this out. We have now added a paragraph in the discussion section to include this important reference (see lines 671 to 679).

The other big issue with this manuscript is that the overall presentation of the data are not straight forward and the authors do not provide adequate experimental details. Throughout the results, discussion and methodology are included. For example, on lines 367-68 of the Results the authors write, “Object location was always pseudorandomly assigned in case there was a bias for location within the arena.” This should have been in the methods section. Relatedly, were the novel objects counterbalanced across subjects? How was exploration scored? By one person or validated across experimenters? Were scorers blinded to which object was novel versus familiar during the test phase? Were they blinded to experimental condition? For the sake of good scientific rigor, these methodological details should be reported.

Thank you. In response to this concern that has been raised by the other two reviewers as well, we have now moved some explanations from the “Results” to the “Methods” section. We also included in the revision details on the protocol and the scoring methodology. Novel objects were always counterbalanced across subjects. Exploration was scored as described in the “Methods” section. Two people scored the videos; one was blind to the novel and familiar objects. There was no significant inter-rater variability (see lines 240 to 257 and 265-6).

Other issues that the authors may want to consider addressing include:

On lines 113-116 the authors state that, “most tasks used to evaluate pattern separation have involved contextual or spatial manipulation.” While behavioral tasks alone cannot be used to evaluate pattern separation (see point above), I invite the authors to look at the work of Toner et al., 2009; Reagh and Yassa, 2014; Reagh et al., 2016; Johnson et al., 2017; Stark & Stark 2017; and others that have examined the discrimination of objects in the context of memory.

Thank you for pointing us at these references. Except for the work of Johnson et al (2017), the rest of the tasks were evaluated in human subjects, which makes it difficult to compare them with the rodent tasks. The work by Johnson et al is quite new, and was not published when the MS was originally written. We have now included this reference. However, in light of the attention to the hippocampus and the dentate gyrus as a structure that disambiguates representations in general, still the majority of the tasks used to evaluate this process in rodents do involve some kind of contextual manipulation. We have now changed the phrase to “with the exception of a few studies (e.g. (Johnson et al., 2017), most tasks used to evaluate the behavioural outputs thought to result from discrimination of overlapping representations in rodents have involved some kind of contextual or spatial manipulation” (lines 77 to 80).

2. On lines 312-313 the authors write, “In the original spontaneous object recognition (SOR) task (Ennaceur and Aggleton, 1997; Warburton et al., 2000)...” These are the wrong citations. The details of the “original SOR task” should be cited as Ennaceur & Delacour, 1988.

Thanks, we have modified the references.

3. Several abbreviations were not defined prior to being used: SOR (defined on line 312) used first on line 212, SLC (used first on line 214).

Thank you. This was corrected.

4. In methods, the authors write, “The objects for the SOR task were made of two different smaller objects, except for the extra-similar condition in which they were made by three even smaller objects.” How much smaller? What are the dimensions? It would be helpful if photos of objects were included.

Thank you for pointing this out, we have now included photos of the objects in the “Methods section” (see Fig. 1). The objects used for the extra-similar condition were actually not smaller than the ones used for the other conditions, this was a mistake and it was corrected. See also our response to Reviewer#2.

5. Along similar lines to point 4, the authors should discuss the caveat of using multiple objects as features, and assuming that the conjunction of several small objects is perceived as a unitary larger object. Another, and perhaps preferable approach is to construct the objects from LEGO blocks to manipulate similarity as done previously (e.g., Bartko et al., 2007; Johnson et al., 2017).

This is an interesting point. However, we did not find any indication that the rats did not treat compound objects as units. If they had treated features separately, vehicle-injected rats would have discriminated objects in the extra-similar condition, which did not happen in any of the two experiments in which we used this version of the SOR task. In fact, if they had treated features separately, we would have expected the animals not to divide exploration time equally between the 3 compound objects during the sample phase in the similar or extra-similar conditions. We would have expected them to spend more time exploring the non-similar object that does not share any of the features with the other two, because it would have been the oddest one as it has been observed for perirhinal lesions in the simultaneous oddity task (Bartko et al, 2007, Learn. Mem.). This did not happen for any of the experiments performed in this study. In addition, while LEGO-constructed objects offer some versatility when trying to manipulate the similarity between them, they could also cause more interference, as the texture would be the same between the different objects. In fact, it has been shown that merely the fact that an object is built with LEGO can cause interference with another LEGO object that is not particularly similar (Bartko, 2010, Neurpsychologia). Junk object features offer different textures and curvy shapes that are not present in LEGO-based objects. However, we acknowledged now in the revision that many other studies have used tasks that involve LEGO compound objects and they obtained similar results (see lines 209 to 216).

Editor

Synthesis Statement for Author (Required):

Although I found the response to the concerns raised by the reviewers thorough and convincing, I was concerned that there were several instances in which a concern was addressed only in the letter, with the clarification never making its way into the manuscript. This was likely exacerbated by the fact that the line references in the response to reviewers are nearly always inaccurate, but I still believe that there are several important points raised (and addressed) in the letter, but not in the manuscript.

By way of example, here is a non-comprehensive list:

- The discussion on page 2 regarding BDNF-ASO reducing steady-state levels at the time of the sample phase.

- Page 4-5: Addressing the reviewer's confusion regarding ODN concentration

- Page 5: The discussion of hr-BDNF administration and Arc expression enhancement

- Page 10: Clarifying the misunderstanding regarding exploration time differences across groups.

I ask that you submit a revised manuscript and response letter that clearly indicates exactly where each point was addressed in the manuscript. If the authors do not wish to change the manuscript in response to a reviewer's concern, please let me know your reason for doing so.

Thank you for this input. As the initial response was for the appeal, some of the responses did not go into the manuscript. We have now included the changes in the text and indicated the corresponding lines in the revised version that includes the tracked changes.

I also note that the reviewers commented on some organizational issues regarding the figures and tables. While it seems that the figures have improved considerably, I still found the tables difficult to follow at first glance. The caption should describe all components of the tables. For example, it's not immediately intuitive what the left-most “Fig n” column depicts. It should be directly stated what statistical comparison was used to generate the p-values depicted in Table III.

We are sorry the tables were unclear. We have now clarified in the table captions all the elements appearing in the tables. We also included more information on the statistical analysis performed to obtain the p values in Table III and the comparisons made.

For Figure 7C, I suggest writing out the full words for “C”, “S”, and “D”, as there appears to be sufficient space. If not, please clearly define these abbreviations.

Thank you for this suggestion. We have written the full words and uploaded a new version of the figure.
